# Interventions to Promote Healthy Meals in Full-Service Restaurants and Canteens: A Systematic Review and Meta-Analysis

**DOI:** 10.3390/nu13041350

**Published:** 2021-04-18

**Authors:** Floriana Mandracchia, Lucia Tarro, Elisabet Llauradó, Rosa Maria Valls, Rosa Solà

**Affiliations:** 1 Functional Nutrition, Oxidation, and Cardiovascular Diseases Group (NFOC-Salut), Healthy Environment Chair, Facultat de Medicina i Ciències de la Salut, Universitat Rovira i Virgili, 43201 Reus, Spain; floriana.mandracchia1@urv.cat (F.M.); lucia.tarro@urv.cat (L.T.); rosamaria.valls@urv.cat (R.M.V.); rosa.sola@urv.cat (R.S.); 2 Institut d’Investigació Sanitaria Pere Virgili, 43204 Reus, Spain; 3 Hospital Universitari Sant Joan de Reus, 43204 Reus, Spain

**Keywords:** out-of-home eating, menu choice, restaurant-based interventions, family, restaurant, food-service, food behavior

## Abstract

Out-of-home eating is increasing, but evidence about its healthiness is limited. The present systematic review and meta-analysis aimed to elucidate the effectiveness of full-service restaurant and canteen-based interventions in increasing the dietary intake, food availability, and food purchase of healthy meals. Studies from 2000–2020 were searched in Medline, Scopus, and Cochrane Library using the PRISMA checklist. A total of 35 randomized controlled trials (RCTs) and 6 non-RCTs were included in the systematic review and analyzed by outcome, intervention strategies, and settings (school, community, workplace). The meta-analysis included 16 RCTs (excluding non-RCTs for higher quality). For dietary intake, the included RCTs increased healthy foods (+0.20 servings/day; 0.12 to 0.29; *p* < 0.001) and decreased fat intake (−9.90 g/day; −12.61 to −7.19; *p* < 0.001), favoring the intervention group. For food availability, intervention schools reduced the risk of offering unhealthy menu items by 47% (RR 0.53; 0.34 to 0.85; *p* = 0.008). For food purchases, a systematic review showed that interventions could be partially effective in improving healthy foods. Lastly, restaurant- and canteen-based interventions improved the dietary intake of healthy foods, reduced fat intake, and increased the availability of healthy menus, mainly in schools. Higher-quality RCTs are needed to strengthen the results. Moreover, from our results, intervention strategy recommendations are provided.

## 1. Introduction

The change in modern living due to urbanization and globalization [1] and the lack of sufficient free time to dedicate to home cooking have increased families’ consumption of daily meals out of the home [2]. Restaurants, schools, workplace canteens and food stores providing prepared meals are the preferred food services by both children and adult populations [3,4].

Consequently, eating out of home is associated with a unhealthy diet [5] due to the lower consumption of fruits and vegetables [6]. Furthermore, comparisons of the nutritional profile of foods have shown that meals prepared out of the home are higher in energy density, fat and sodium and lower in calcium and fiber than foods prepared at home [7]. Thus, consumers of out-of-home meals may report important long-term health implications, such as obesity [8] and related chronic diseases [9]. In this regard, people are paying more attention to the healthiness of food when eating out of home [10], demanding higher-quality meals from food businesses that have the responsibility to provide them according to consumers’ necessities [11].

For instance, potential strategies for the promotion of healthier meals could be the improvement of the nutritional quality of food in terms of energy, fat and sodium [12], the reduction of portion sizes in meals [13] and the provision of nutritional labels [14]. The lack of nutritional information on menus, known as the consumer “nutritional knowledge gap”, could hinder people’s healthy eating intentions when they are eating out of home [15].

However, the literature on the most effective interventions to improve consumers’ diet when they are eating out of home is still scarce. Moreover, most nutrition interventions are set in fast-food and chain restaurants mainly placed in urban areas [16], leaving little evidence about independent restaurants and potential intervention strategies [17,18].

Another aspect is identifying suitable solutions for different population targets [19], such as children, adolescents and adults, and in different environments, such as restaurants [18], schools [20] and workplace canteens [21].

Thus, the aim of the present systematic review and meta-analysis is to elucidate the effectiveness of full-service restaurant- and canteen-based interventions targeting children, adolescents and adults in increasing the availability, purchase and intake of healthy meals.

## 2. Materials and Methods

This systematic review has been registered in the International Prospective Register of Systematic Reviews (PROSPERO) with the registration number CRD42019117411. The results of the included articles are reported according to the Preferred Reporting Items for Systematic Reviews and Meta-Analysis (PRISMA) guidelines [22]. The PRISMA 2009 checklist is presented in Table S1.

### 2.1. Search Strategy

Three electronic databases were searched: Medline, Scopus, and Cochrane Library. Search filters were used in all three databases to limit the results to the “2000–2020” publication time range and English, Spanish, and Italian language articles. For the abstract and full-text screening of the articles, the Rayyan QCRI web-based software platform [23] was used to better manage the high volume of retrieved articles. Searches were conducted using the following keywords: “intervention” AND “controlled” AND “restaurant” OR “canteen” OR “food-service” AND “meal” OR “dietary intake” OR “food availability” OR “food purchase” OR “menu”.

The Population, Intervention, Comparison and Outcomes (PICOS) criteria (Table 1) were used to define the research question of the present systematic review [24].

### 2.2. Screening

Initial screening of the title, abstract, keywords and publication type was conducted by two reviewers independently (F.M.; L.T.). Full-text screening of potentially relevant studies was independently performed by the same two reviewers (F.M.; L.T.) based on the inclusion and exclusion criteria, and disagreements were resolved by a third reviewer (E.L.). Final doubts about the eligibility of a particular study were resolved through discussion between the three reviewers for further confirmation and consensus.

### 2.3. Inclusion and Exclusion Criteria

The inclusion criteria used for the selection of eligible articles in this review were (a) controlled trials, with or without random assignment, published from 2000 to 2020 to focus the search on healthy eating interventions in full-service restaurants and canteens, conducted in contemporary circumstances; (b) English, Spanish or Italian language articles; (c) articles describing full-service restaurant and canteen-based interventions aimed at improving menu offerings and increasing the offerings and demand for healthier meals as the primary or secondary outcome; (d) trials that included a control group (CG) that did not receive the intervention; and (e) trials that presented both pre- and postintervention measurements of the intervention group (IG) and the CG and the *p*-values of the difference between groups.

Articles were excluded when (1) they did not fulfill the abovementioned criteria; (2) they used the pretest condition as the CG; or (3) the authors of the article were not able to give further details about the intervention results when personally asked by the authors of the present paper.

### 2.4. Data Extraction and Management

The following data were extracted from the included intervention studies: (1) study design and type of intervention; (2) setting; (3) country; (4) population; (5) population age; (6) duration of the intervention; (7) outcome; (8) measurement tool; (9) results; and (10) intervention strategies.

If necessary, further information about the results was collected by emailing the corresponding authors [25,26,27,28,29], especially when it was not possible to deduce the information directly from tables and figures.

The extracted results included mean changes from baseline to postintervention or follow-up and significant differences between groups in changes from pre- to postintervention. For each variable examined in between-group comparisons, differences were considered significant at *p*-values ≤0.05.

### 2.5. Data Synthesis

For a better evaluation of intervention effectiveness, the included interventions were divided according to the following: (1) outcome category (dietary intake, food availability and food purchase); (2) strategies applied (consumer- and/or establishment-based); and (3) intervention setting (school, community, and workplace) reflecting the age of the target population, i.e., children and/or adults.

Moreover, the included interventions were classified similarly to previous studies as follows [30,31]: (1) effective, when all the measured variables indicated a statistically significant change from baseline to post assessment in favor of the IG compared to the CG; (2) partially effective, when some variables included in the study changed significantly favoring the IG and any variable changed favoring the CG; and (3) not effective, when any significant changes occurred or when a change favoring the CG occurred. For the interpretation of the final effectiveness of the systematic review, an intervention was considered effective when the corresponding study reported it to be totally and/or partially effective.

### 2.6. Outcomes

The included studies focused on different outcomes, which were grouped into three major categories, as described previously. Specifically, (1) the dietary intake outcome category referred to the increase in the study population’s consumption of healthier meals, which, according to the World Health Organization (WHO) recommendations, requires the consumption of more fruits and vegetables, the limitation of the consumption of saturated and trans fats and sugar and salt, and a balanced energy intake [32]; (2) the food availability outcome category referred to the change in the offerings of healthy and/or unhealthy food items (in terms of quality and quantity) in restaurants and canteens, which represents one of the highest-impact interventions for changing the population’s dietary behavior [33]; and (3) the food purchase outcome category referred to the change in consumers’ food selection towards the selection of healthier food options offered in restaurants and canteens, which is directly related to the increase in the availability of such options to satisfy consumers’ demands [34].

### 2.7. Data Analysis

The meta-analysis was performed with Review Manager 5.4.1 and STATA 16.1 (StataCorp. 2019. Stata Statistical Software: Release 16. StataCorp LLC, College Station, TX, USA) when at least three of the included intervention studies presented similar outcome variables and units of measure. Meta-analysis was performed including both RCTs and non-RCTs, and then it was repeated by excluding non-RCTs to assure higher quality results. Studies were analyzed with a random effect model when the heterogeneity of the studies was evaluated over 75% by the I^2^ statistic, with the results expressed as odds ratios (ORs). When the heterogeneity was <75%, the fixed effects model was used, and the results were expressed as the risk ratio (RR) [35]. Intervention studies that presented the same measurement units and outcomes were analyzed in subgroups of studies. If the SD, SE or 95% CI values were not available in the original articles, the intervention studies were not included in the meta-analysis. A *p*-value of ≤0.05 was considered statistically significant.

### 2.8. Risk of Bias and Quality Criteria

The risk of bias and quality assessment of the included intervention studies was performed using the standardized framework of the Quality Assessment Tool for Quantitative Studies Dictionary developed for the Effective Public Health Practice Project [36]. Each included intervention study was evaluated as weak, moderate or strong for six of the eight specific categories: selection bias, study design, confounders, blinding, data collection methods, and withdrawals and dropouts. Then, the overall quality of the studies was appraised based on a 3-point rating scale including strong (no weak ratings), moderate (one weak rating) or weak (two or more weak ratings).

## 3. Results

### 3.1. Results of the Search

**Figure 1:**
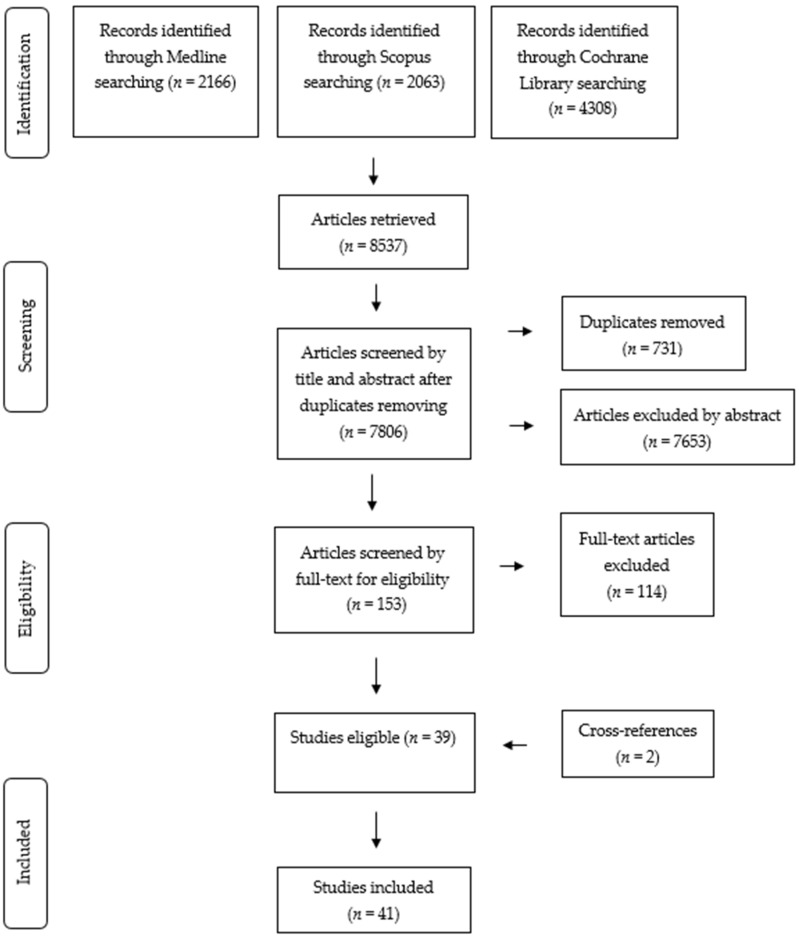
Preferred Reporting Items for Systematic Reviews and Meta-Analyses (PRISMA) 2009 flow diagram for the systematic review of the article selection process.

A total of 8537 articles were retrieved from the search of the Medline, Cochrane Library and Scopus databases (Figure 1). A total of 731 duplicates were removed, resulting in 7806 articles for title and abstract screening. Of these, 7653 were excluded because they were irrelevant for the present review by title and abstract screening. The remaining 153 articles were selected for further full-text screening according to the inclusion criteria. Following the screening, an additional 114 articles were excluded for not fulfilling the inclusion criteria. A total of 39 English-language articles were evaluated as eligible for inclusion, together with 2 articles resulting from cross-reference searching, for a total of 41 articles finally included in the present systematic review. The detailed general characteristics of the included studies are shown in Table 2, and the results on the mean pre–post intervention changes in the IG and CG are shown in Table S2.

### 3.2. General Characteristics of the Included Intervention Studies

The 41 included interventions (Table 2) were based on different study designs: 35 were RCTs [25,26,27,28,29,37,38,39,40,41,42,43,44,45,46,47,48,49,50,51,52,53,54,55,56,57,58,59,60,61], and 6 were non-RCTs [62,63,64,65,66,67]. A total of 3 studies consisted of 4-arm parallel-group conditions [27,57,64] and 4 studies had a 3-arm parallel-group intervention [26,28,49,67].

The included interventions were set in 12 different countries. Most of them were performed in the United States (*n* = 20) [26,27,28,37,38,40,48,51,52,55,56,60,61,63,65,66,67,68,69,70] and Australia (*n* = 11) [25,39,41,43,45,46,47,58,59,62,71], while the other studies were conducted in Lebanon [29], Brazil [44], Denmark [50], Malaysia [42], Scotland [53], Belgium [49], Mexico [72], Ecuador [54], Ireland [64], and the UK [57].

### 3.3. Settings of the Included Studies

The included studies took place in different settings. Twenty-four RCT and 5 non-RCT studies were applied in school settings, specifically in primary and secondary schools [25,27,29,38,41,42,44,46,47,48,49,53,54,55,57,60,63,65,66,68,69,71] or in a childcare service center [39,43,58,59,62,67,72]. Then, 8 RCTs were conducted in community settings, including 1 intervention in sporting clubs [45]; 2 interventions in restaurants [26] and/or food stores [40]; 4 interventions in after-school programs in churches, communities and schools [37,51,52,70]; and 1 intervention in recreation centers, including corner stores, wholesalers and carry-out restaurants [56]. Four interventions, including 3 RCTs and 1 non-RCT, were conducted in workplace settings [28,50,61,64] (Table 2).

### 3.4. Samples of the Included Studies

The total sample size of the 41 included studies was 35,638 participants (IG: 18,988; CG: 16,650) (Table 2). In particular, there were 16,824 participants in dietary intake interventions (children, school chefs and employees), 9361 participants in food availability interventions (children, school chefs, customers and club members), and 20,019 participants in food purchase interventions (children, employees, club members and customers). The study samples were varied and stratified in terms of sample size (from 28 to 3908 people) and age (children and adults, as reflected by the different settings).

### 3.5. Intervention Duration

The applied interventions lasted from 1 week to 3 years (Table 2). In particular, in the dietary intake outcome category, 16 interventions lasted less than one year [27,29,43,44,50,51,52,53,57,58,63,64,65,66,68,69] and 6 interventions lasted up to one year [49,54,55,56,59,67]. In the food availability outcome category, 6 interventions lasted less than one year [38,40,41,42,43,44] and 8 interventions lasted up to one year [37,39,45,46,47,48,62,72]. In the food purchase outcome category, 13 interventions lasted less than one year, [25,26,27,28,29,40,60,61,65,66,69,70,71] and 3 interventions lasted up to one year [45,46,56].

### 3.6. Intervention Type

The intervention type was based on the strategies used. Each intervention applied different consumer-based and establishment-based strategies to achieve the evaluated outcome (Table 3). In particular, 3 consumer-based strategies were used to provide support, information and education (defined as a, b, and c) to consumers to improve their healthy food choices. Nine establishment-based strategies (defined as d to l) were applied for the improvement of the nutrition environment, including implementing menus offering healthier options and increasing the knowledge of restaurants and food service staff about healthy nutrition. Based on the strategies used in effective interventions, strategy recommendations were derived according to the outcome and setting applied (Table 4).

### 3.7. Outcomes

The 41 included interventions were analyzed for one or more of the three outcomes identified and mentioned above: (1) 22 interventions (17 RCTs and 5 non-RCTs) aimed to improve customers’ dietary intake regarding the consumed food and beverage items and the nutritional composition of food in terms of micro- and macronutrients [27,29,43,44,49,50,51,52,53,54,55,56,57,58,59,63,64,65,66,67,68,69]; (2) 14 interventions (13 RCTs and 1 non-RCT) aimed to increase healthy food offerings on menus [37,38,39,40,41,42,43,44,45,46,47,48,62,72]; and (3) 16 interventions (14 RCTs and 2 non-RCTs) aimed to increase the population’s healthy food purchases [25,26,27,28,29,40,45,46,56,60,61,65,66,69,70,71] (Table 2).

### 3.8. Dietary Intake Outcome Category

A total of 11 of the 22 interventions targeting dietary intake outcome [27,29,43,52,57,58,59,65,66,68,69] presented results on the population’s food and beverage intake. On the other hand, 2 interventions [64,67] presented results on the population’s nutrient intake, and 9 interventions assessed both food and beverage intake and nutrient intake [44,49,50,51,53,54,55,56,63] (Table S2).

Among these 22 interventions focused on dietary intake, (a) 1 intervention effectively improved children’s dietary intake for all the measured variables by increasing healthy food items and decreasing unhealthy ones [52]; (b) 17 interventions were partially effective in changing the population’s dietary intake of some of the evaluated healthy or unhealthy menu items and nutrients (sugar, fat, saturated fat, energy and sodium) [27,29,43,49,50,51,53,56,58,59,63,64,67,68,69], of which 2 studies were partially effective only in some of the evaluated IGs [27,49]; and (c) 4 interventions reported no effectiveness for any of the evaluated variables [44,57,65,66] (Table 2).

One effective intervention was set in a community setting as an after-school program and lasted 9 months [52]. Among the 17 partially effective interventions, 2 were implemented in workplace settings, 2 in community settings and 13 were implemented in school settings, and they lasted from 3 weeks to 30 months [27,29,43,49,50,51,53,56,58,59,63,64,67,68,69].

### 3.9. Food Availability Outcome Category

A total of 3 RCTs of the 14 interventions that targeted food availability outcome [41,46,47] presented results of the analysis of menu offerings in school canteens (no “red” or banned food items and >50% “green” food items). The other 11 interventions (10 RCTs and 1 non-RCT) [37,38,39,40,42,43,44,45,48,62,72] presented food availability results in relation to the increase or decrease in healthy items (fruits and vegetables, unsweetened beverages, water, whole grains, etc.) or unhealthy items (high-fat, high-energy, high-sugar and high-sodium foods) on the menus of restaurants and canteens, and 3 of these studies also evaluated changes in the availability of nutrients [42,44,48] (Table S2).

Among these 14 interventions focused on food availability, 3 interventions effectively improved food availability for all the measured variables by increasing menu offerings of healthy food and beverage items and decreasing the offerings of unhealthy ones in the IG compared to the CG [41,43,46].

On the other hand, 7 interventions were partially effective by significantly changing the availability of only some of the evaluated variables, which were healthy/unhealthy food items offered on the menu, in favor of the IG [38,39,42,45,47,48,72]. Furthermore, 4 intervention studies reported no positive changes for any of the evaluated variables or reported negative changes for some variables in favor of the IG [37,40,44,62] (Table 2).

The 3 food availability interventions that were totally effective [41,43,46] were conducted in school settings, namely primary and secondary schools, and lasted from 6 to 14 months. On the other hand, among the 7 partially effective interventions, 1 was implemented in the community and 6 were implemented in schools, and they lasted from 1 week to 3 years [38,39,42,45,47,48,72].

### 3.10. Food Purchase Outcome Category

Among the 16 interventions targeting food purchase outcomes [25,26,27,28,29,40,45,46,56,60,61,65,66,69,70,71], (a) 13 presented results on food and beverage items purchased in restaurants and food service establishments by customers [26,27,28,29,40,45,56,60,61,65,66,69,71], (b) 1 intervention [46] presented results on healthy purchases in terms of food nutrient content, and (c) 2 interventions presented results on both food items and nutrient content [25,70] (Table S2).

Among these 16 interventions focused on food purchases, 3 were totally effective: 2 interventions effectively improved the population’s purchase of healthy food items and beverages [45,66], and the other 3-arm intervention reported an increase in “green” food items purchased in only one of the IGs [28]. Another 9 interventions were partially effective in changing the population’s food purchase of some of the evaluated healthy or unhealthy menu items, also according to their nutrient content (sodium, sugar, energy) [25,27,29,46,56,60,61,65,70], and the other 4 interventions reported no effectiveness for any of the evaluated variables [26,40,69,71] (Table 2).

Between the 3 totally effective interventions, 1 was implemented in a workplace setting, namely, hospital canteens [28], 1 in the school [66] and 1 a community setting, namely, sporting club canteens [45]; they lasted from 5 weeks to 2.5 years.

On the other hand, of the 9 partially effective interventions, 6 were conducted in school settings, 2 in community settings and 1 in a workplace setting, and they lasted from 5 weeks to 14 months [25,27,29,46,56,60,61,65,70].

### 3.11. Results of the Meta-Analysis

A total of 16 RCTs [27,41,43,44,46,47,49,50,51,52,53,55,56,58,59,69] and 3 non-RCTs [63,64,67] comprising 20,897 participants in total were included in the meta-analysis for the evaluation of dietary intake and food availability outcomes. For dietary intake outcome, 9 studies (8 RCTs and 1 non-RCT) were included to analyze the increase in servings/day of healthy food items [27,43,51,52,56,58,59,63,69], 5 studies (3 RCTs and 2 non-RCTs) for the increase in the intake of fiber g/day [50,51,55,64,67], 5 studies (3 RCTs and 2 non-RCTs) for the decrease of nutrients g/day [49,50,51,64,67], 5 studies (3 RCTs and 2 non-RCTs) for the decrease in energy percentage (%E) deriving from fat [49,50,53,64,67], and 7 studies (4 RCTs and 3 non-RCTs) for the decrease of daily caloric intake [44,51,55,56,63,64,67]. For food availability outcomes, 3 RCT studies were included to evaluate the proportion of school canteen menus offering healthier food items by reducing unhealthy items (red or banned items) [41,46,47]. Any intervention study for the food purchase outcome could be included in the present meta-analysis because of the lack of data to be compared. With the exception of food availability, where the studies included presented similar interventions (I^2^ statistic = 43%), the forest plots of dietary intake outcome presented high heterogeneity (I^2^ statistic ≥ 90%). Thus, for dietary intake outcome, the analysis was conducted with randomized and nonfixed effect models, and the results are expressed as weighted mean differences with 95% CIs between the pre- and postintervention values of both the IG and CG. For food availability outcomes, meta-analysis was performed by pooling risk ratios (RRs) using the Mantel–Haenszel method.

#### 3.11.1. Dietary Intake Meta-Analysis

**Figure 2:**
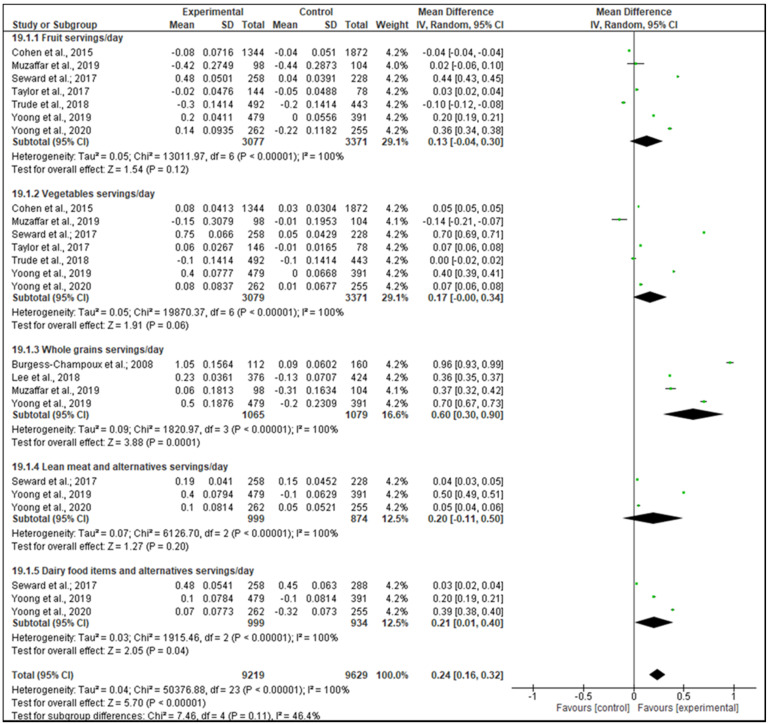
Forest plot of the effectiveness of increasing the dietary intake of healthy food items (servings/day), according to the included intervention studies (RCTs and non-RCTs).

**Figure 3:**
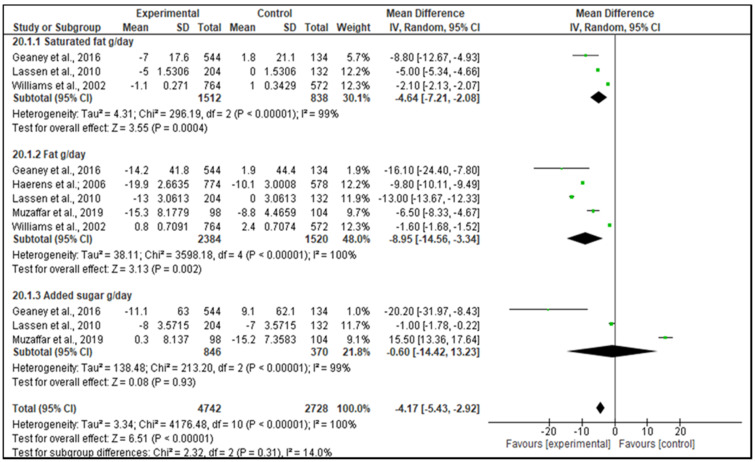
Forest plot of the effectiveness of decreasing the dietary intake of saturated fat, fat and added sugar nutrients (g/day) according to the included intervention studies (RCTs and non-RCTs).

For the dietary intake outcome, the included intervention studies (RCTs and non-RCTs) were effective in increasing +0.24 servings/day of healthy food groups in favor of the IG (95% CI, 0.16 to 0.32; *p* < 0.001; Figure 2), including fruit, vegetables, whole grains, lean meat, and alternatives (poultry, fish, eggs, tofu, seeds, and legumes), dairy food items and alternatives (milk, yogurt, cheese). Specifically, +0.60 servings/day of whole grain (95% CI, 0.30 to 0.90; *p* < 0.001; Figure 2) and +0.21 servings/day of dairy food items and alternatives (95% CI, 0.01 to 0.40; *p* = 0.04; Figure 2) significantly increased in favor of the IG. Moreover, when non-RCTs were excluded from the meta-analysis, the effectiveness was also confirmed (Figure S1). An increase of +0.50 g/day of fiber was also observed in favor of the IG for the analyzed intervention studies (95% CI, 0.08 to 0.92; *p* = 0.02; Figure S1). However, when non-RCTs were excluded from the meta-analysis, the effectiveness was not confirmed (Figure S1). Furthermore, a positive decrease of −4.17 g/day of nutrients such as saturated fat, fat and added sugar (95% CI, −5.43 to −2.92; *p* < 0.001; Figure 3) occurred, favoring the IG. Specifically, −4.64 g/day saturated fat (95% CI, −7.21 to −2.08; *p* < 0.001; Figure 3) and −8.95 g/day fat (95% CI, −14.56 to −3.34; *p* = 0.002; Figure 3) significantly decreased in favor of the IG. However, when non-RCTs were excluded from the meta-analysis, only fat intake could be assessed since at least 3 studies were included and the effectiveness was confirmed (Figure S1*).*

On the other hand, no effectiveness was observed in the overall effect size for the intervention studies aimed at reducing the percentage of caloric intake derived from fat (%E/day) (dietary intake, −3.50; 95% CI, −7.24 to 0.24; *p* = 0.07; Figure S1). Moreover, these results were confirmed when excluding non-RCTs from the meta-analysis (Figure S1).

Furthermore, a significant increase in the daily total caloric intake of +25.59 kcal/day (95% CI, 10.80 to 40.37; *p* < 0.001; Figure S1) was observed in favor of the CG and remained significant in the CG when non-RCTs were excluded from the analysis (Figure S1).

#### 3.11.2. Food Availability Meta-Analysis

**Figure 4:**
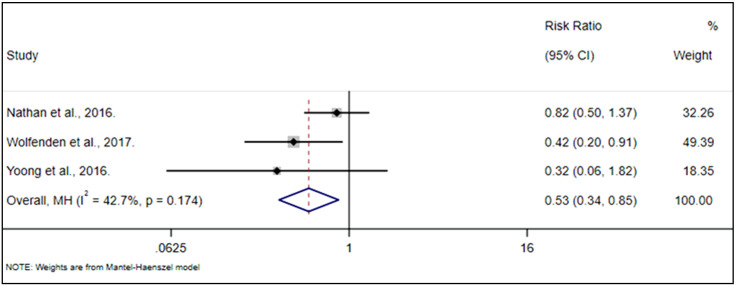
Forest plot of the relationship between the effectiveness of the included interventions (RCTs) and the risk for schools to offer unhealthy items on canteen menus.

Regarding the food availability outcome, the included interventions effectively reduced the risk of intervention schools offering unhealthy items on canteen menus by 47%, labeled red or banned food items and beverages (RR 0.53; 95% CI, 0.34 to 0.85; *p* = 0.008; I2 = 43%; Figure 4).

### 3.12. Quality Assessment Results

Based on the risk of bias and quality assessment of the included studies, all of the studies were of weak quality, and blinding was not used in any study due to the nature of the intervention (Table S3). Although all 41 studies presented a strong study design, the majority of them had weak selection bias [25,26,27,28,37,40,41,42,45,48,49,51,53,54,56,61,63,66,67,69,71,72], weak confounders [25,26,27,28,29,38,39,40,41,42,43,45,48,51,53,57,58,59,60,61,63,64,66,67,68,69,71], and weak data collection methods [25,26,27,28,29,37,38,39,40,42,44,45,46,47,48,50,51,52,53,54,55,56,59,60,61,62,66,67,68,69,70,71,72]. Additionally, regarding withdrawals and dropouts, studies presented mixed results with 16 weak [26,27,37,38,40,42,48,50,51,53,55,61,62,65,67,72], 12 moderate [43,47,49,52,54,56,58,59,60,64,66,69], and 13 strong [25,28,29,39,41,44,45,46,57,63,68,70,71] (Table S3).

Since all the studies included in the systematic review had weak quality, the meta-analysis was performed considering RCT and non-RCT intervention studies together, and it was repeated by excluding non-RCTs to assure results with higher quality.

## 4. Discussion

The present systematic review included 41 interventions, 35 RCTs and 6 non-RCTs, and of these, 16 RCTs and 3 non-RCTs were included in the meta-analysis. Eligible interventions were full-service restaurants and canteen-based interventions aimed at increasing dietary intake, food availability, and food purchases in different settings, such as schools, workplaces, and communities. The results from the present systematic review showed that restaurant- and canteen-based interventions are effective in improving healthy dietary intake and food availability, mainly in the school setting, with a beneficial impact on children. However, there is partial evidence for the improvement of food purchases, and more evidence is needed about workplaces and community settings as full-service restaurants. Moreover, when the meta-analysis was performed without considering non-RCT studies, the results were confirmed in dietary intake for increasing healthy food intake and in the reduction of fat intake.

The results are discussed considering systematic review and meta-analysis outcomes because meta-analysis contributes to evaluating the effectiveness of this type of intervention, and systematic review allows us to review the characteristics of interventions with effective results.

The included interventions in the meta-analysis demonstrated effectiveness in increasing the intake of healthy food items (whole grains, dairy products and alternatives) and nutrients such as fiber [27,43,50,51,52,55,56,58,59,63,64,67,69] mainly in children, demonstrating that schools are a favorable environment for the promotion of healthy dietary intake. Furthermore, an increase in daily caloric intake occurred in favor of the CG [44,51,55,56,63,64,67], and effectiveness was observed for decreasing the consumption of other nutrients such as saturated fat and fat in the IG [49,50,51,64,67]. For food availability outcome, the intervention studies included in the meta-analysis were also demonstrated to be effective in reducing the risk, for the intervention schools, of offering unhealthy foods and beverages on canteen menus [41,46,47].

For interventions in the dietary intake outcome category, the present results showed effectiveness mainly in school settings, which was the preferred setting for interventions targeting these outcomes. When targeting children, an important factor to be considered in nutrition interventions is food presentation in terms of color and smell, which should be appetized to trigger food selection and consumption. Thus, repeated exposure to healthier foods presented in attractive ways could help children become more accustomed to and consume it [73]. Focusing on adults, changing dietary habits to achieve a healthier lifestyle is made more difficult by the perceived barriers, such as: lack of cooking skills and willpower; time scarcity; the need to give up one’s favorite foods [74]; and social, cultural and economic conditions [75]. However, although the evidence about workplace settings is very limited in the present review, workplace interventions have the potential to change consumers’ dietary behavior through the working lifespan [76]. Long-term workplace interventions for approximately one year evidenced an improvement in dietary change among the participants [77], while the included studies in this systematic review lasted less than one year. However, it is important to highlight that published evidence and its quality in workplace programs are suboptimal; thus, this conclusion needs to be verified with high-quality interventions [77].

From the present results, regarding the intervention strategies applied to improve dietary intake, the implementation of establishment-based interventions is different in the three evaluated settings. Specifically, the strategies that showed higher effectiveness in schools were the addition of healthier menu options combined with on-site support, training for the school canteen staff, performance monitoring and feedback reports (Table 4). However, in the community setting, including after school programs and recreation centers, the provision of monetary incentives, rewards, and recognition for the participating food service are effective, while these methodologies are ineffective in schools.

According to the interventions in the food availability outcome category, none of them were set in workplaces, and little evidence resulted in the community setting [45], whereas effectiveness was reported in the school setting [38,39,41,42,43,45,46,47,48,72]. In schools, regarding the intervention strategies applied for food availability outcomes, the involvement of the participants’ families, namely students and their parents in school-based interventions, through invitations to meetings, activities and the distribution of information letters, was the most effective consumer-based strategy [41,46,72]. Similarly, in a recent review focusing on family-based interventions to improve children’s diets, the family involvement strategy through the provision of information, advice and monitoring was also reported to be effective in improving the food environment of school canteens, demonstrating that parents are an important component when children are targeted [78].

Children’s improvements in food availability are important because their adherence persists in adulthood, whereas unhealthy food availability reinforces children’s preference for nutrient-poor and ultra-processed foods [79]. The increase in healthy food availability in school settings is directly correlated with healthy food purchases, with the final aim of changing children’s dietary intake [80].

On the other hand, the implementation of healthier food availability in the community setting is more difficult due to the barriers stakeholders encounter, such as the lack of demand by customers and the increased cost associated with healthy fresh foods with a short shelf life [81,82,83], but financial support and resources such as guidelines and training from established associations could help achieve such improvements [81]. Thus, future interventions aimed at increasing the availability of healthier food options in community settings should also target an increase in consumers’ demands for healthy meals, as well as assure food services of the low risk of changes in their profits [84].

For the interventions in the food purchase outcome category, partial effectiveness was reported mainly in schools through the implementation of multiple consumer- and establishment-based strategies, including the involvement of participants’ families [25,27,29,46,60,65,66]; thus, family certainly has a good influence on children’s food selection [85].

On the other hand, little evidence about effective strategies in community and workplace settings was apparent in the present systematic review; however, in community settings such as restaurants and food stores, the provision of information and communication to consumers may not be enough to achieve behavior changes such as the selection of healthier food options [26,40], whereas multiple strategies targeting changes in the food environment could be fundamental for improving customers’ food purchases [45].

Moreover, effective consumer- and establishment-based strategies were derived from the included interventions to develop methodological recommendations, by outcome and setting, for the implementation of future restaurant and canteen-based interventions (Table 4). There were some limitations in the present systematic review and meta-analysis. First, the lack of randomized controlled studies in workplace and community settings, such as full-service restaurants, limited the evidence about the adult population and the evaluation of the interventions’ effectiveness. Second, the exclusion of fast-foods and chain restaurants in this systematic review and meta-analysis limited the generalizability of the results to other out-of-home settings, but it allowed us to provide specific recommendations for full-service restaurants and canteens. Third, the lack of enough evidence for the different community settings included, such as after-school programs, restaurants, sporting clubs, and recreation centers, made it difficult to detect differences in intervention strategies. Fourth, none of the included studies were set in low-income countries because of the intervention gap in the literature about middle- and low-income countries [86], limiting the inclusivity of a wider target population. Fifth, in the meta-analysis, the wide heterogeneity of the included studies in terms of outcomes and units of measure, and the huge quantity of different outcomes included, as well as the lack of specific numerical data in the articles, made it difficult to compare interventions and reduced the interventions included. Finally, the quality of most of the included studies was assessed to be of weak quality since the majority had no blinding, poor data collection methods, selection bias or confounders.

## 5. Conclusions

In conclusion, restaurant- and canteen-based interventions demonstrated effectiveness in the improvement of healthy food intake and in the reduction of fat intake and in increasing healthy menu availability, mainly in school settings. For food purchases, a systematic review showed that interventions could be partially effective in improving healthy foods. However, higher-quality RCTs are needed to strengthen the results. Moreover, intervention strategy recommendations were provided for each outcome assessed to increase the effectiveness of restaurant-based interventions implemented.

## Figures and Tables

**Table 1:** PICOS criteria used to define the research question.

Criteria	Description
Population	Restaurant and canteen consumers (children and adults) and their staff.
Intervention	Restaurant- and canteen-based interventions concerning the promotion of healthy meals.
Comparison group	Comparison Group as a CG receiving any intervention.
Outcomes	Improvement in the promotion of healthy foods offered in restaurants and canteens; increase in the offer and the demand for healthy meals.
Setting	Restaurants and canteens.

**Table 2:** Characteristics of the included intervention studies in restaurants and food service establishments.

Study	Study Design and Type of Intervention	Setting	Country	Study Samples	Age	Duration ^1^	Effectiveness	Between Groups Significance	Between Groups No Significant Changes
Ayala et al.; 2017.	Cluster RCT; 3-arm restaurant-based intervention.	Community (restaurants)	USA	8 restaurants (Menu-plus IG *n* = 4; Menu-only CG *n* = 4) and weekend dinner customers (*n* = N/A).	≥12 y	2 m	Food purchase x	−	Weekly sales of new child menus ($/week).
Anderson et al.; 2005.	RCT; school-based nutrition education intervention.	School	Scotland	4 schools (IG *n* = 2; CG *n* = 2) and 129 students (IG *n* = 64; CG *n* = 65).	6–7 y and 10–11 y	9 m	Dietary intake ✓x	↑ Fruit (g).	Vegetables (g), total F&V (g), energy (kJ), % energy as fat/carbohydrate/protein, starch (g), sucrose (g).
Beets et al.; 2016.	RCT; multistep adaptive intervention.	Community (after-school program)	USA	9 schools (IG *n* = 3; CG *n* = 6), 4 churches (IG *n* = 3; CG *n* = 1), 7 communities (IG *n* = 4; CG *n* = 3) and 1765 students (IG *n* = 895; CG *n* = 870).	6–12 y	12 m	Food availability x	↑ F&V (days), ↑ dips (days); ↓ desserts (days), ↓ salty unflavored snacks (days), ↓total sweetened beverages (days), ↓ 100% fruit juice (days).	Dairy unsweetened snacks (days), dairy sweetened snacks (days), salty flavored snacks (days), unsweetened cereals (days), sugar-sweetened cereals (days), water, unflavored milk (days).
Bogart et al.; 2014.	RCT; multicomponent intervention.	School	USA	10 schools (IG *n* = 5; CG *n* = 5) and 3039 students (IG *n* = 1515; CG *n* = 1524).	±12–13 y	5 w	Food purchase ✓x	↑ All lunches (servings), ↑ free/reduced lunch (servings), ↑ full-price lunches (servings); ↑fruit servings during intervention; ↓ snack sales.	Fruit and vegetable servings postintervention.
Cohen et al.; 2014.	RCT; single-component intervention.	School	USA	8 schools (IG *n* = 4; CG *n* = 4) and 2746 students (IG *n* = 1550; CG *n* = 1196).	6–12 y	1 w	Food availability ✓x	↑ % of days offering WG (lunch).	WG and RG (options/breakfast and lunch), % of days offering WG (breakfast), % of days offering RG (breakfast and lunch).
Cohen et al.; 2015	RCT; 4-arm chef and choice architecture school-based intervention.	School	USA	14 schools (Chef IG-A *n* = 2; Smart Café IG-B *n* = 4; Chef plus Smart Café IG-C *n* = 2; CG *n* = 6) and 2638 students (Chef IG-A *n* = 379; Smart Café IG-B *n* = 651; Chef plus Smart Café IG-C *n* = 672; CG *n* = 936).	8–16 y	7 m (long-term intervention)	Dietary intake ✓x(IG-A, IG-C);x (IG-B).	↑ cups of fruits (IG-A), ↑ cups of vegetables (IG-A, IG-C), ↑ % of vegetables (IG-A, IG-C).	% Entrée, % cups of fruit (IG-B, IG-C), Cups of vegetable (IG-B), % of fruit (IG-A, IG-B, IG-C), % of vegetables (IG-B).
							Food purchase ✓x (IG-A, IG-B, IG-C)	↑ % of students selecting fruit and vegetables (IG-A, IG-B, IG-C).	% of students selecting entrée (*p* = N/A).
Delaney et al.; 2017.	Cluster RCT; consumer behavior intervention.	School	Australia	10 schools (IG *n* = 5; CG *n* = 5) and 2714 students (IG *n* = 1144; CG *n* = 1570).	5–12 y	2 m (+2 m follow-up)	Food purchase ✓x	↑ Green menu items (%); ↓ energy (kJ), ↓ sodium (mg), ↓ saturated fat (g), ↓ red menu items (%).	Sugar (g).
Giles et al.; 2012.	RCT; environmental and policy change intervention.	Community (after-school programs)	USA	20 after-school programs (IG *n* = 10; CG *n* = 10) and 145 students (IG *n* = 62; CG *n* = 83).	±8 y	6 m	Food purchase ✓x	↑ water (ounces), ↑ frequency of water served/day; ↓ kcal from beverages served/day.	100% juice (ounces and frequency of service), milk (ounces and frequency of service).
Grady et al.; 2020.	RCT; web-based menu-planning intervention.	School (childcare centers)	Australia	54 childcare centers (IG *n* = 27; CG *n* = 27) and 54 menu planners (IG *n* = 27; CG *n* = 27).	3–6 y	12 m	Food availability ✓x	↑ Fruit (servings), ↑ meat and alternatives (servings); ↓ discretionary foods (unhealthy) (times/day).	Servings of: vegetables, cereals and breads, dairy and alternatives.
Habib-Mourad et al.; 2014.	Pilot cluster RCT; multicomponent intervention.	School	Lebanon	8 schools (IG *n* = 4; CG *n* = 4) and 374 students (IG *n* = 193; CG *n* = 181).	9–11 y	3 m	Food purchase ✓x	↓ Chips (%, *n*), ↓ chocolate (%, *n*), ↓ soft drinks (%, *n*).	Sweetened beverages (*p* = N/A), croissant (%, *n*), manoushe (%, *n*).
							Dietary intake ✓x	↓ Chips (%, *n*), ↓ soft drinks (%, *n*).	Chocolate (%, *n*), sweetened drinks (%, *n*), fruit (%, *n*), sandwich (%, *n*).
Haerens et al.; 2006.	RCT; 3-arm environmental and computer-tailored intervention.	School	Belgium	15 schools (parental involvement IG-A *n* = 5; intervention alone IG-B *n* = 5; CG *n* = 5) and 2840 students (IG-A *n* = 1226; IG-B/CG *n* = N/A).	±13 y	2 y	Dietary intake ✓x (girls); x (boys).	↓ Fat (g) (girls), ↓ %E from fat (girls).	Fat (g) (boys), %E from fat (boys), pieces of fruit (boys and girls), soft drinks (glass) (boys and girls), water (glass) (boys and girls).
Kenney et al.; 2015.	Cluster RCT; school cafeteria-based intervention.	School	USA	10 schools (IG *n* = 5; CG *n* = 5) and 1599 students (IG *n* = 725; CG *n* = 874).	6–18 y	3 w	Dietary intake ✓x	↑ Water (ounces), ↑ % students consuming free water; ↓ % students consuming 100%juice, ↓ % students consuming sugar-sweetened beverages.	% students consuming milk, % students consuming other beverages.
Lassen et al.; 2010.	Cluster RCT; participatory and empowerment-based intervention.	Workplace	Denmark	8 workplaces (IG *n* = 5; CG *n*= 3) and 168 employees (IG *n* = 102; CG *n* = 66).	±42 y	6 m	Dietary intake ✓x	↑ Fiber (g/10 MJ), %E in carbohydrate; ↓ fat (g/day), ↓ saturated fat (g/day), ↓ fat (%E/day), ↓ cake and sweets (g/day and g/10 MJ).	Energy (kJ), protein (%E/day), added sugar (g/day and g/10 MJ), fiber (g/day), F&V (g/day and g/10 MJ), potatoes (g/day and g/10 MJ).
Lee et al.; 2018.	Cluster RCT; multilevel intervention.	Community (after-school programs)	USA	20 after-school programs (IG *n* = 10; CG *n* = 10) and 400 students (IG *n* = 188; CG *n* = 212).	≥5 y	9 m	Dietary intake ✓ (for on-site food services)	↑ whole grains (servings), ↑ F&V (servings); ↓ ounces 100% juice, ↓ foods with trans fats (servings), ↓ food and beverage calories (servings).	‒
Martínez-Donate et al.; 2015.	Pilot RCT; food environment restaurant and food store-based intervention.	Community (food stores and restaurants)	USA	14 restaurants (IG *n* = 7; CG *n* = 7), 4 food stores (IG *n* = 2; CG *n* = 2), 721 restaurant customers (IG *n* = 319; CG *n* = 402) and 601 food store customers (IG *n* = 299; CG *n* = 302).	N/A	10 m	Food purchase x	‒	% of restaurant orders, % of food store. purchases.
							Food availability x	‒	Restaurant and food store nutrition environment (NEMS-R NEMS-S).
Morshed et al.; 2016.	Cluster RCT; multilevel obesity-prevention intervention.	School (childcare centers)	Mexico	16 childcare centers (IG *n* = 8; CG *n* = 8) and children (*n* = N/A).	3 y	2 y	Food availability ✓x	↓ Daily grams of fat from milk.	Fruit (servings), vegetables (servings), whole grains (servings), discretionary fat (grams), added sugar (teaspoons).
Muzaffar et al.; 2019.	Cluster RCT; peer education intervention.	Community (after-school programs)	USA	7 school groups (peer-led IG *n* = 4; adult-led CG *n* = 3) and 101 children (IG *n* = 49; CG *n* = 52).	11–14 y	3 m	Dietary intake ✓x	↑ Whole grains (servings).	Total kcal/day, fruits (servings), vegetables (servings), total fat/sugar/fiber/salt (g).
Nathan et al.; 2016.	RCT; multicomponent intervention.	School	Australia	53 schools (IG *n* = 28; CG *n* = 25) and 499 students (IG mean *n* = 232; CG mean *n* = 267).	5–12 y	9 m	Food availability ✓	↑ Menu with no red/banned items, ↑ menu with >50% of green items.	‒
Ochoa-Avilés et al.; 2017.	Cluster RCT; curriculum and environment-based intervention.	School	Ecuador	20 schools (IG *n* = 10; CG *n* = 10) and 1430 students (IG *n* = 702; CG *n* = 728).	12–14 y	28 m	Dietary intake ✓x	↑ F&V (g); ↓ added sugar (g), ↓ unhealthy snacking (g).	Unhealthy snacking at school (proportion difference), breakfast intake (proportion difference), fat (%E/day).
Rosmawati et al.; 2017.	Cluster RCT; school-canteen intervention.	School	Malaysia	16 schools (IG *n* = 8; CG *n* = 8) and 110 food handlers (IG *n* = 52; CG *n* = 58).	18–55 y	6 w (+12 w follow-up)	Food availability ✓x	↑ Milk and milk products (% served food).	% served food: carbohydrate, protein, fat, added sugar, vegetable, fruits, forbidden and not recommended foods, fast foods.
Seward et al.; 2017.	RCT; multistrategy intervention.	School (childcare centers)	Australia	45 childcare centers (IG *n* = 25; CG *n* = 20), canteen cooks (IG *n* = 25; CG *n* = 20), and 243 students (IG *n* = 129; CG *n* = 114).	N/A	6 m	Food availability ✓	Servings of: ↑ vegetables, ↑ fruit, ↑ breads and cereals, ↑ meat and alternatives, ↑ dairy; ↓ discretionary foods (unhealthy).	‒
							Dietary intake ✓x	↑ Vegetables (servings), ↑ fruit (servings).	Servings of: breads and cereals, meat, dairy, discretionary.
Siega-Riz et al.; 2011.	Cluster RCT; school-based intervention.	School	USA	42 schools (IG *n* = 21; CG *n* = 21) and 3908 students (IG *n* = 1964; CG *n* = 1944).	10–14 y	30 m (five school semesters)	Dietary intake ✓x	↑ Fruit (g), ↑ water (g).	Energy (kcal), carbohydrates (g), protein (g), fat (g), fiber (g), grains (g), vegetables (g), legumes (g), sweets (g), sweetened beverages (g), fruit juice (g), fat and whole milk (g), 1% fat milk (g).
Souza et al.; 2013.	Cluster RCT; nutrition educational intervention.	School	Brazil	20 schools (IG *n* = 10; CG *n* = 10), 95 school lunch chefs (IG *n* = 47; CG *n* = 48) aged ±46 years, and students (*n* = N/A).	N/A	7 m	Food availability x	−	kg/child of: sugar, donuts, milky coffee, banana cereals, chocolate cereals, chocolate milk, powdered milk, cake mix.
							Dietary intake x	−	Energy (kcal), carbohydrates (%), lipid (%), protein (%), % energy derived from sugar/sweets/sugary drinks, portions/day of added sugar/sugary drinks/sweets.
Story et al.; 2003.	RCT; multicomponent multicenter intervention.	School	USA	41 schools (IG *n* = 21; CG *n* = 20) and 1700 students (IG/CG *n* = N/A).	7–9 y	3 y	Food availability ✓x	↑ % energy from carbohydrates; ↓ % energy from total fat and saturated fat.	Total calories (kcal), total fat (g), saturated fat (g), protein (g), % energy from protein, carbohydrates (g), total sugars (g), sucrose (g), dietary fiber (g), sodium (mg).
Taylor et al.; 2017.	Pilot RCT; multicomponent intervention.	School	USA	2 schools (IG *n* = 1; CG *n* = 1) and 294 students (IG *n* = 161; CG *n* = 133).	9–10 y	9 m	Dietary intake ✓x	↑ Vegetable (cups).	Fruit (cups).
							Food purchase x	‒	Vegetable (cups), fruit (cups).
Thorndike et al.; 2016.	RCT; 3-arm social norm intervention.	Workplace (hospital cafeteria)	USA	1 hospital and 2672 employees (feedback-only IG-A *n* = 877; feedback-incentive IG-B *n* = 925; CG *n* = 870).	≥18 y	3 m	Food purchase ✓(IG-B); x (IG-A)	↑ Green menu items (%) (IG-B).	Green menu items (IG-A) (%).
Trude et al.; 2018.	Cluster RCT; multilevel and multicomponent intervention.	Community (recreation centers including wholesalers, corner stores and carryout restaurants)	USA	30 recreation center zones (IG *n* = 14; CG *n* = 16) and 401 child–caregiver dyads (IG *n* = 209; CG *n* = 192).	9–15 y	14 m	Dietary intake ✓x	↓ % kcal from sweets (13-15y).	Total daily caloric intake, sugary beverages (kcal), fruit punch (ounces), dietary total sugar (g), dietary sodium (mg), fruit (total cups), vegetable (total cups), fat (servings) (9–15y); % kcal from sweets (9–12y).
							Food purchase ✓x	↑ healthier foods and beverages items per week (9–12y); ↑ unhealthy foods and beverages items per week (9–12y).	Healthy and unhealthy foods and beverages items per week (13–15y).
Warren et al.; 2003.	Pilot RCT; 4-arm school and family-based intervention.	School	UK	3 schools and 218 students (Eat Smart IG-A *n* = 56; Play Smart IG-B *n* = 54; Eat/Play Smart IG-C *n* = 54; Be Smart CG *n* = 54).	5–7 y	5 m	Dietary intake x (IG-A, IG-B, IG-C)	−	Weekly portion frequency of: vegetables, salads, fresh fruit, other fruit, confectionery, crisps (IG-A, IG-B, IG-C).
Webb et al.; 2011.	Pilot RCT; menu labeling intervention.	Workplace (hospital cafeteria)	USA	6 cafeterias (menu board plus poster labeling IG *n* = 2; CG *n* = 2) and 554 customers (IG *n* = 334; CG *n* = 220).	>18 y	2 m	Food purchase ✓x	↑ % target side dishes (healthy), ↑ % target snacks (healthy).	% target entrées (healthy) (data N/A).
Wolfenden et al.; 2015.	Cluster RCT; multicomponent intervention.	Community (sporting clubs)	Australia	85 sporting clubs (IG *n* = 42; CG *n* = 43) and 1394 club members (IG *n* = 689; CG *n* = 705).	±34 y	2.5 y	Food availability ✓x	↑ F&V availability and promotion (%, *n*).	Non sugar-sweetened beverages (%, n).
							Food purchase ✓	↑ F&V (%, *n* items purchased), ↑ no sugar-sweetened beverages (%, *n* items purchased).	‒
Wolfenden et al.; 2017.	RCT; multistrategic intervention.	School	Australia	70 schools (IG *n* = 35; CG *n* = 35) and 509 students (IG mean *n* = 256; CG mean *n* = 253).	5–12 y	12/14 m	Food availability ✓	↑ Menu with no red/banned items, ↑ menu with >50% of green items.	‒
							Food purchase ✓x	↓ Total fat (g).	Energy (kJ), sodium (mg).
Wyse et al.; 2019.	Cluster RCT; online menu choice architecture intervention.	School	Australia	6 schools (IG *n* = 3; CG *n* = 3) and 1938 students (IG *n* = 1203; CG *n* = 735).	4–12 y	4 w	Food purchase x	‒	% lunch orders containing target items (Fruit & Vegetable), % lunch order items that are target items (Fruit & Vegetable).
Yoong et al.; 2016.	RCT; multicomponent intervention.	School	Australia	72 schools (IG *n* = 36; CG *n* = 36) and 426 students (IG mean *n* = 216; CG mean *n* = 210).	5–12 y	12 m	Food availability ✓x	↓ % of red items in the menu.	Menus with no red or banned foods and beverages, menus with >50% of green items, % of amber, and green items.
Yoong et al.; 2019.	Cluster RCT; food service multistrategy intervention.	School (childcare centers)	Australia	28 childcare centers (IG *n* = 15; CG *n* = 13), 395 students (IG *n* = 220; CG *n* = 175) and 28 cooks (IG *n* = 15; CG *n* = 13).	2–5 y	6 m	Dietary intake ✓x	↑ Vegetables (servings), ↑ whole grain cereals (servings), ↑ meat/meat alternatives (servings).	Fruit (servings), dairy/dairy alternatives (servings).
Yoong et al., 2020.	Cluster RCT; web-based menu-planning intervention.	School (childcare centers)	Australia	35 childcare centers (IG/CG *n* = N/A) and 220 children for baseline dietary data observation (IG *n* = 112; CG *n* = 108).	2–6 y	12 m	Dietary intake ✓x	↑ Fruit (servings), ↑ dairy and alternatives (servings); ↓ cereals and bread (servings), ↓ discretionary foods (unhealthy) (times consumed).	Vegetables (servings), meat and alternatives (servings).
Bell et al.; 2014.	Non-RCT; implementation intervention.	School (childcare centers)	Australia	431 childcare centers (IG *n* = 240; CG *n* = 191) and 153 children (IG *n* = 79; CG *n* = 74).	3–6 y	20 m (+ 5-m follow-up)	Food availability x	↑ Vegetable (servings); ↓ high-fat/salt/sugar food (items), ↓sweetened beverages (items); ↓ fruit (servings).	‒
Bogart et al.; 2011.	Pilot non-RCT; obesity-prevention and peer leader advocacy intervention.	School	USA	2 middle schools (IG *n* = 1; CG *n* = 1) and 399 students (IG/CG *n* = N/A).	±13 y	5 w	Food purchase ✓	↑ Fruits (% students served); ↑ healthy entrées (% students served).	‒
							Dietary intake x	‒	Soda (%students drink), sports/fruit drinks (% students drink).
Burgess-Champoux et al.; 2008.	Pilot non-RCT; multicomponent school-based intervention.	School	USA	2 schools (IG *n* = 1; CG *n* = 1) and 150 parent/child pairs (IG *n* = 67; CG *n* = 83).	±10 y	3 m	Dietary intake ✓x	↑ WG (servings), ↑ fiber (g); ↓ RG (servings).	Energy (kcal).
Geaney et al.; 2016.	Cluster non-RCT; 4-arm workplace-based intervention.	Workplace	Ireland	4 workplaces (Education IG-A *n* = 1; Environment IG-B *n* = 1; Combined IG-C *n* = 1; CG *n* = 1) and 517 employees (Education IG-A *n* = 107; Environment IG-B *n* = 71; Combined IG-C *n* = 272; CG *n* = 67).	18–64 y	±7 9 months (intervention+ follow-up)	Dietary intake ✓x	↓ Salt (g) (IG-C), ↓ saturated fat (g/day for IG-A, IG-C, and %E for IG-B, IG-C), ↓ total sugars (g) (IG-B).	Salt intake (g) (IG-A, IG-B), total energy (kcal), total fat (g/day and %E), saturated fat (g) (IG-B), %E saturated fat (IG-A), total sugars (g) (IG-A, IG-C), fiber (g) (IG-A, IG-B, IG-C).
Quinn et al.; 2018.	Non-RCT; behavioral economics-based choice architecture intervention.	School	USA	11 schools (IG *n* = 6; CG *n* = 5) and 2245 students (IG *n* = 1026; CG *n* = 1219).	11–18 y	7 m	Dietary intake x (among students who selected)	↑ proportion students consuming fruit (including juice), ↑ fruit items consumed (excluding juice), ↑ vegetables items consumed (including potatoes) in favor of the CG.	Proportion students consuming: fruit (including/excluding juice), vegetables (including/excluding potatoes), low-fat milk; mean number of: fruit (including/excluding juice), vegetables (including/excluding potatoes), low-fat milk.
							Food purchase ✓x	↑ proportion students selecting fruit (including/excluding juice); ↑ fruit items (including/excluding juice).	Proportion students selecting: vegetables (including/excluding potatoes), low-fat milk; mean number of: vegetables (including/excluding potatoes), low-fat milk.
Williams et al.; 2002.	Cluster non-RCT; 3-arm nutrition education and food service intervention.	School (childcare centers)	USA	9 childcare centers (nutrition education IG-A *n* = 3; safety education IG-B *n* = 3; CG *n* = 3) and 1296 students (IG/CG *n* = N/A).	2–5 y	20 m	Dietary intake ✓x (results of IG-A and IG-B are presented together)	↓ Saturated fat (g), ↓ fat and saturated fat (% kcal), ↑ iron (mg), ↑ magnesium (mg).	Fat (g), kcal, cholesterol (mg), protein (g), fiber (g), calcium (mg), zinc (mg), Vitamin A and Folic Acid and Vitamin B12 (microgram), Vitamin E and C (mg), riboflavin (mg).

The included studies in the present systematic review are sorted in the following table by RCTs and non-RCTs and by alphabetical order. N/A: not available; F&V: fruit and vegetable; WG: whole grain; RG: refined grain. ✓: effective; x: not effective; ✓x: partially effective. ^1^: duration in weeks (-w), months (-m) or years (-y).

**Table 3:** Effectiveness of the strategies used in the included intervention studies.

Setting	Studies	Outcome Categories					
		Food Availability		Dietary Intake		Food Purchase	
		Consumer-Based Strategies	Establishment-Based Strategies	Consumer-Based Strategies	Establishment-Based Strategies	Consumer-Based Strategies	Establishment-Based Strategies
**School**	Anderson et al.; 2005.			✓x a, b, c	✓x d		
	Bogart et al.; 2014.					✓x a, b, c	✓x d, h, i, k
	Cohen et al.; 2014.		✓x e, f, g				
	Cohen et al., 2015.				✓x (IG-A, C) x (IG-B) IG-A: d, f, g; IG-B and IG-C: d, f, g, i		✓x (IG-A, B, C) d, f, g, i
	Delaney et al.; 2017.						✓x d, i, h, k
	Grady et al., 2020		✓x e				
	Habib-Mourad et al.; 2014.			✓x a, b, c	✓x d	✓x a, b, c	✓x d
	Haerens et al.; 2006			✓x (girls), x (boys) IG-A, B: a, b IG-A: c	✓x (girls), x (boys) IG-A, B: d, e, f, g, h		
	Kenney et al.; 2015.			✓x a	✓x d		
	Morshed et al.; 2016.	✓x a, b, c	✓x d, e, f, g, k				
	Nathan et al.; 2016.	✓ c	✓ d, e, f, g, h, j				
	Ochoa-Avilés et al.; 2017.			✓x a, b, c	✓x e, f		
	Rosmawati et al.; 2017.		✓x e, f, g				
	Seward et al.; 2017.		✓ d, f, g, h		✓x d, f, g, h		
	Siega-Riz et al.; 2011.			✓x a, b, c	✓x d		
	Souza et al.; 2013.		x e, f		x e, f		
	Story et al.; 2003.		✓x e, f, g, j				
	Taylor et al.; 2017.			✓x a, b, c	✓x d, e, j	x a, b, c	x d, e, j
	Warren et al.; 2003.			x IG-A and C: a, b, c; IG-B: b, c	x IG-A, B, C: j		
	Wolfenden et al.; 2017.	✓ c	✓ d, e, f, g, h, j			✓x c	✓x d, e, f, g, h, j
	Wyse et al.; 2019.						x g, i
	Yoong et al.; 2016.		✓x d, e, g, h				
	Yoong et al.; 2019.				✓x e, f, g, h		
	Yoong et al., 2020.				✓x e, f, g, h		
	Bell et al.; 2014.	x a, c	x e, f, g, h, j				
	Bogart et al.; 2011.			x a, b	x d, f, i, j	✓ a, b	✓ d, f, i, j
	Burgess-Champoux et al.; 2008.			✓x a, b, c	✓x d, f, g, h		
	Quinn et al.; 2018.				x d, e, f, g, i, j		✓x d, e, f, g, i, j
	Williams et al.; 2002.				✓x IG-A, B: d, f		
**Community**	Ayala et al.; 2017.					x a	x d, f, g, h, i
	Beets et al.; 2016.		x d, f, g				
	Giles et al.; 2012					✓x c	✓x d, e, f, g, h, j
	Lee et al.; 2018.			✓ a, b, c	✓ d, e, f, g, h, j		
	Martínez-Donate et al.; 2015.	x a, b	x d, g, i			x a, b	x d, g, i
	Muzaffar et al.; 2019.			✓x a, b	✓x e, j		
	Trude et al.; 2018.			✓x a, b, c	✓x f, i, j	✓x a, b, c	✓x f, i, j
	Wolfenden et al.; 2015.	✓x a	✓x d, e, f, g, h, i, j, l			✓ a	✓ d, e, f, g, h, i, j, l
**Workplace**	Lassen et al.; 2010.			✓x a, b	✓x d, e, f		
	Thorndike et al.; 2016.					x(IG-A), ✓(IG-B) IG-A and B: a	✓(IG-B) IG-B: l
	Webb et al.; 2011.					✓x a	✓x i, k
	Geaney et al.; 2016.			✓x (IG-A, B, C) IG-A and C: a, b	✓x (IG-A, B, C) IG-A: g, k; IG-B: d, g, i, l; IG-C: d, g, i, k, l		

The included studies in the present systematic review are sorted in the following table by RCTs and non-RCTs and by alphabetical order. ✓: Effective; **x**: Not effective; ✓x: Partially effective. Consumer-based strategies: (a) provision of promotional/educational materials in the form of leaflets, posters, manuals, emails and messages directed to consumers; (b) organization of workshops/lessons/meetings/activities for customers; and (c) participants’ family involvement through letters, meetings, and activities in school canteen-based interventions. Establishment-based strategies: (d) implementation of a menu with healthier options and limitation of the unhealthier ones, including meal portion-size control and nutrient-content limitations; (e) provision of promotional/educational materials in the form of leaflets, posters, manuals, emails and messages directed to the restaurant and canteen staff; (f) training of the restaurant and canteen managers and chefs; (g) professional on-site and remote support; (h) performance monitoring and feedback reports for the restaurants and canteens; (i) point-of-purchase strategic food positioning, attractive packaging, prompts, menu inserts, and symbols; (j) monetary incentives/rewards/recognition for the participating restaurants and canteens; (k) food labeling information (i.e., traffic light system), and (l) price discounts for customers. The strategies shown in this table are derived from the recommendations in Table 4.

**Table 4:** Strategy recommendations derived from effective interventions included in the systematic review.

Setting	Outcome Categories		
	Food Availability	Dietary Intake	Food Purchase
**School**	The involvement of the students’ families, as a consumer-based strategy, together with the application of multiple establishment-based strategies, seemed to be effective in improving food availability in the school setting.	The application of consumer-based strategies together with the implementation of a menu with healthier options and limitation of the unhealthier ones, applied alone or in combination with other establishment-based strategies, seemed to be effective in improving dietary intake in the school setting. On the other hand, the provision of monetary incentives/rewards/recognition for the participating school canteen was not effective.	The application of consumer-based strategies together with the implementation of a menu with healthier options and limitation of the unhealthier ones, applied alone or in combination with other establishment-based strategies, seemed to be effective in improving food purchases in the school setting.
**Community**	No recommendation can be provided about both consumer- and establishment-based strategies.	The application of consumer-based strategies, together with establishment-based strategies such as the provision of monetary incentives/rewards/recognition for the participating restaurant or canteen, seemed to be effective in improving dietary intake in the community setting.	The application of multiple establishment-based strategies, including monetary incentives/rewards/recognition for the participating restaurant or canteen, seemed to be effective in improving food purchases in the community setting.
**Workplace**	Outcome not evaluated.	The application of consumer-based strategies together with the implementation of a menu with healthier options and limitation of the unhealthier ones, as an establishment-based strategy, seemed to be effective in improving dietary intake in the workplace setting; however more evidence is needed.	No recommendation can be provided about both consumer- and establishment-based strategies.

These recommendations are based on the interventions included in the present systematic review, as shown in Table 3.

## Supplementary Materials

The following are available online at https://www.mdpi.com/article/10.3390/nu13041350/s1, Table S1: PRISMA 2009 Checklist; Table S2: Included intervention study results; Table S3: Quality of the included intervention studies; Figure S1: Forest plots of the included intervention meta-analysis.

## Abbreviations

CG: Control Group; F&V: Fruit and Vegetable; IG: Intervention Group; N/A: Not Available; RCT: Randomized Controlled Trials; RG: Refined Grain; WG: Whole Grain.
