# Supplementary material for: Interventions to Promote Healthy Meals in Full-Service Restaurants and Canteens: A Systematic Review and Meta-Analysis
Source: Nutrients. 2021 Apr 18;13(4):1350. doi: 10.3390/nu13041350 (PMC8073122; doi:10.3390/nu13041350)
Supplement: Supplementary file 1 [file nutrients-13-01350-s001.zip › Figure S1_Forest plots of the included intervention meta-analysis_.pdf]

Figure S1: Forest plots of the included intervention meta-analysis.

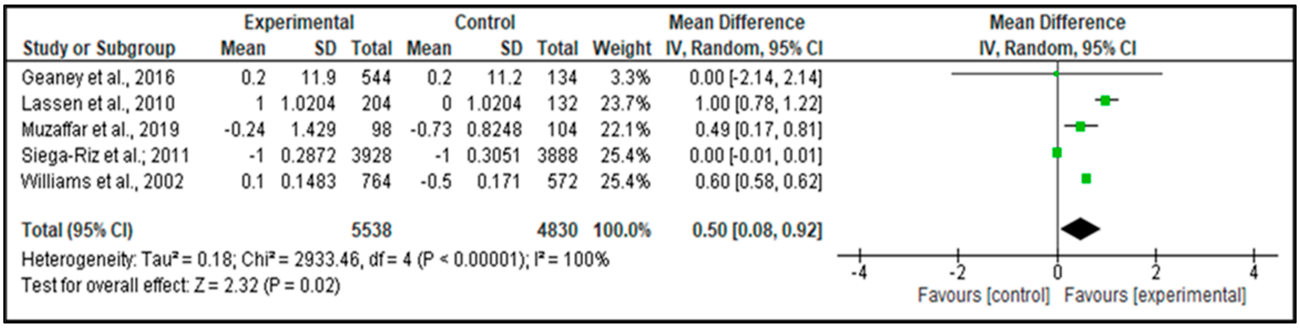

Forest plot of the effectiveness of increasing the dietary intake of fiber (g/day) according to the included intervention studies (RCTs and non-RCTs).

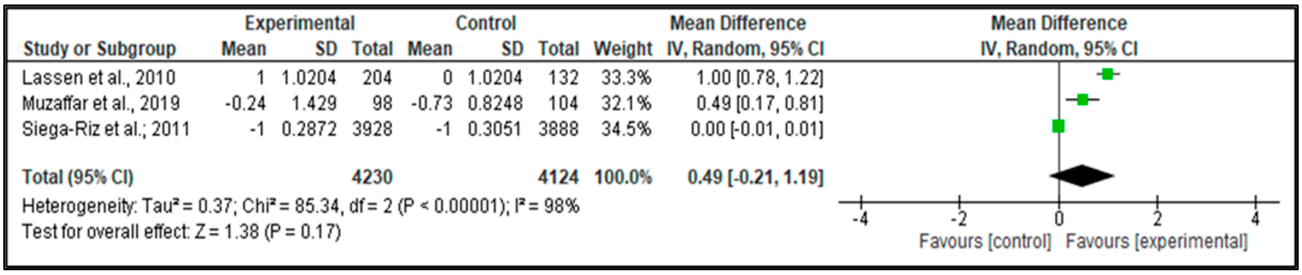

Forest plot of the effectiveness of increasing the dietary intake of fiber (g/day) according to the included intervention studies (RCTs only).

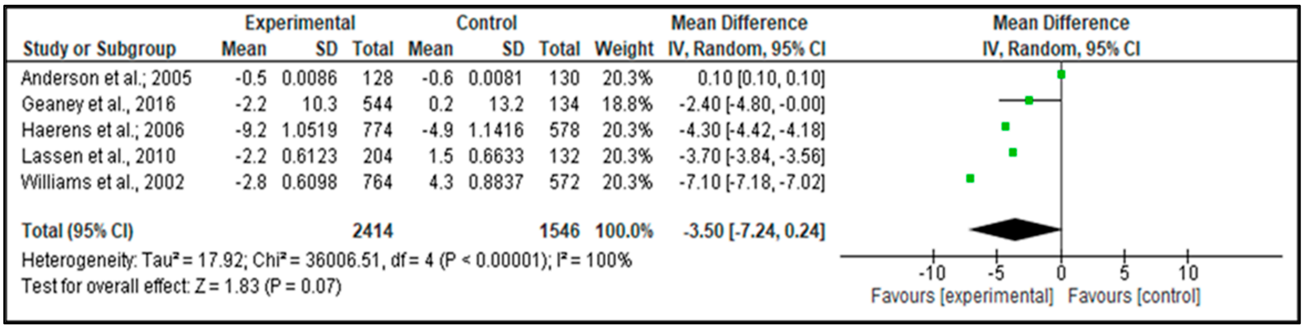

Forest plot of the effectiveness of decreasing the percentage of energy derived from fat (%E/day) according to the included intervention studies (RCTs and non-RCTs).

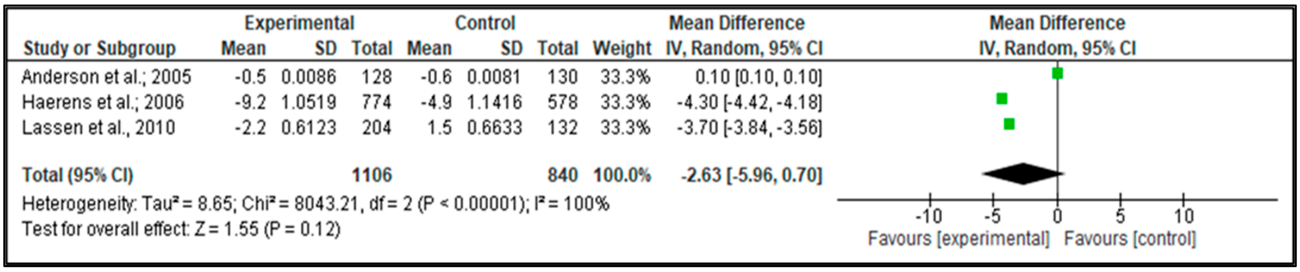

Study or Subgroup	Experimental Mean	Experimental SD	Experimental Total	Control Mean	Control SD	Control Total	Weight	Mean Difference IV, Random, 95% CI
Anderson et al.; 2005	-0.5	0.0086	128	-0.6	0.0081	130	33.3%	0.10 [0.10, 0.10]
Haerens et al.; 2006	-9.2	1.0519	774	-4.9	1.1416	578	33.3%	-4.30 [-4.42, -4.18]
Lassen et al., 2010	-2.2	0.6123	204	1.5	0.6633	132	33.3%	-3.70 [-3.84, -3.56]
**Total (95% CI)**			**1106**			**840**	**100.0%**	**-2.63 [-5.96, 0.70]**

Heterogeneity: Tau² = 8.65; Chi² = 8043.21, df = 2 (P < 0.00001); I² = 100%
Test for overall effect: Z = 1.55 (P = 0.12)

Forest plot of the effectiveness of decreasing the percentage of energy derived from fat (%E/day) according to the included intervention studies (RCTs only).

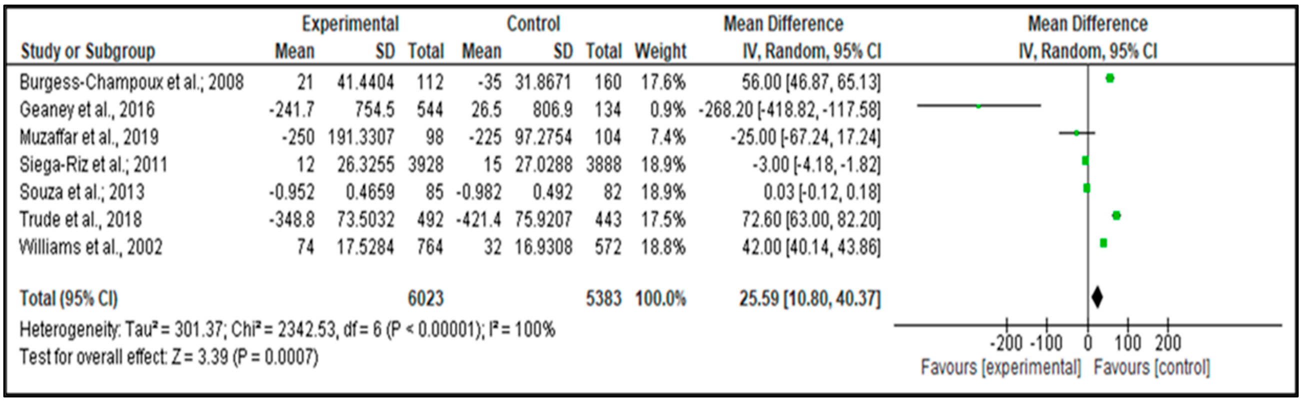

Study or Subgroup	Experimental Mean	Experimental SD	Experimental Total	Control Mean	Control SD	Control Total	Weight	Mean Difference IV, Random, 95% CI
Burgess-Champoux et al.; 2008	21	41.4404	112	-35	31.8671	160	17.6%	56.00 [46.87, 65.13]
Geaney et al., 2016	-241.7	754.5	544	26.5	806.9	134	0.9%	-268.20 [-418.82, -117.58]
Muzaffar et al., 2019	-250	191.3307	98	-225	97.2754	104	7.4%	-25.00 [-67.24, 17.24]
Siega-Riz et al.; 2011	12	26.3255	3928	15	27.0288	3888	18.9%	-3.00 [-4.18, -1.82]
Souza et al.; 2013	-0.952	0.4659	85	-0.982	0.492	82	18.9%	0.03 [-0.12, 0.18]
Trude et al., 2018	-348.8	73.5032	492	-421.4	75.9207	443	17.5%	72.60 [63.00, 82.20]
Williams et al., 2002	74	17.5284	764	32	16.9308	572	18.8%	42.00 [40.14, 43.86]
**Total (95% CI)**			**6023**			**5383**	**100.0%**	**25.59 [10.80, 40.37]**

Heterogeneity: Tau² = 301.37; Chi² = 2342.53, df = 6 (P < 0.00001); I² = 100%
Test for overall effect: Z = 3.39 (P = 0.0007)

Forest plot of the effectiveness of decreasing the total daily caloric intake (kcal/day) according to the included intervention studies (RCTs and non-RCTs).

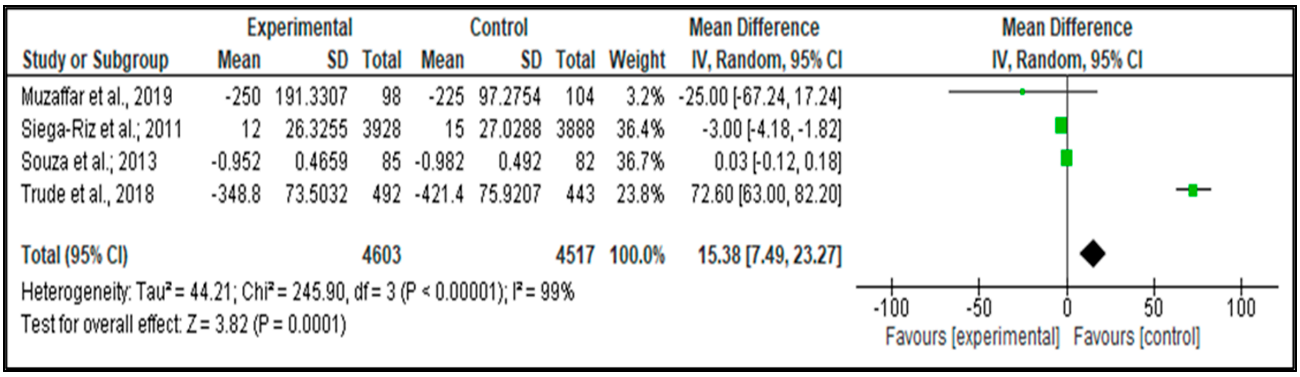

Study or Subgroup	Experimental Mean	Experimental SD	Experimental Total	Control Mean	Control SD	Control Total	Weight	Mean Difference IV, Random, 95% CI
Muzaffar et al., 2019	-250	191.3307	98	-225	97.2754	104	3.2%	-25.00 [-67.24, 17.24]
Siega-Riz et al.; 2011	12	26.3255	3928	15	27.0288	3888	36.4%	-3.00 [-4.18, -1.82]
Souza et al.; 2013	-0.952	0.4659	85	-0.982	0.492	82	36.7%	0.03 [-0.12, 0.18]
Trude et al., 2018	-348.8	73.5032	492	-421.4	75.9207	443	23.8%	72.60 [63.00, 82.20]
**Total (95% CI)**			**4603**			**4517**	**100.0%**	**15.38 [7.49, 23.27]**

Heterogeneity: Tau² = 44.21; Chi² = 245.90, df = 3 (P < 0.00001); I² = 99%
Test for overall effect: Z = 3.82 (P = 0.0001)

Forest plot of the effectiveness of decreasing the total daily caloric intake (kcal/day) according to the included intervention studies (RCTs only).

Study or Subgroup	Experimental Mean	Experimental SD	Experimental Total	Control Mean	Control SD	Control Total	Weight	Mean Difference IV, Random, 95% CI
**22.1.1 Fruit servings/day**								
Cohen et al., 2015	-0.08	0.0716	1344	-0.04	0.051	1872	4.4%	-0.04 [-0.04, -0.04]
Muzaffar et al., 2019	-0.42	0.2749	98	-0.44	0.2873	104	4.2%	0.02 [-0.06, 0.10]
Seward et al.; 2017	0.48	0.0501	258	0.04	0.0391	228	4.4%	0.44 [0.43, 0.45]
Taylor et al., 2017	-0.02	0.0476	144	-0.05	0.0488	78	4.4%	0.03 [0.02, 0.04]
Trude et al., 2018	-0.3	0.1414	492	-0.2	0.1414	443	4.4%	-0.10 [-0.12, -0.08]
Yoong et al., 2019	0.2	0.0411	479	0	0.0556	391	4.4%	0.20 [0.19, 0.21]
Yoong et al., 2020	0.14	0.0935	262	-0.22	0.1182	255	4.4%	0.36 [0.34, 0.38]
**Subtotal (95% CI)**			**3077**			**3371**	**30.4%**	**0.13 [-0.04, 0.30]**
Heterogeneity: Tau² = 0.05; Chi² = 13011.97, df = 6 (P < 0.00001); I² = 100%								
Test for overall effect: Z = 1.54 (P = 0.12)								
**22.1.2 Vegetables servings/day**								
Cohen et al., 2015	0.08	0.0413	1344	0.03	0.0304	1872	4.4%	0.05 [0.05, 0.05]
Muzaffar et al., 2019	-0.15	0.3079	98	-0.01	0.1953	104	4.2%	-0.14 [-0.21, -0.07]
Seward et al.; 2017	0.75	0.066	258	0.05	0.0429	228	4.4%	0.70 [0.69, 0.71]
Taylor et al., 2017	0.06	0.0267	146	-0.01	0.0165	78	4.4%	0.07 [0.06, 0.08]
Trude et al., 2018	-0.1	0.1414	492	-0.1	0.1414	443	4.4%	0.00 [-0.02, 0.02]
Yoong et al., 2019	0.4	0.0777	479	0	0.0668	391	4.4%	0.40 [0.39, 0.41]
Yoong et al., 2020	0.08	0.0837	262	0.01	0.0677	255	4.4%	0.07 [0.06, 0.08]
**Subtotal (95% CI)**			**3079**			**3371**	**30.4%**	**0.17 [-0.00, 0.34]**
Heterogeneity: Tau² = 0.05; Chi² = 19870.37, df = 6 (P < 0.00001); I² = 100%								
Test for overall effect: Z = 1.91 (P = 0.06)								
**22.1.3 Whole grains servings/day**								
Lee et al., 2018	0.23	0.0361	376	-0.13	0.0707	424	4.4%	0.36 [0.35, 0.37]
Muzaffar et al., 2019	0.06	0.1813	98	-0.31	0.1634	104	4.3%	0.37 [0.32, 0.42]
Yoong et al., 2019	0.5	0.1876	479	-0.2	0.2309	391	4.3%	0.70 [0.67, 0.73]
**Subtotal (95% CI)**			**953**			**919**	**13.0%**	**0.48 [0.24, 0.71]**
Heterogeneity: Tau² = 0.04; Chi² = 513.87, df = 2 (P < 0.00001); I² = 100%								
Test for overall effect: Z = 3.98 (P < 0.0001)								
**22.1.4 Lean meat and alternatives servings/day**								
Seward et al.; 2017	0.19	0.041	258	0.15	0.0452	228	4.4%	0.04 [0.03, 0.05]
Yoong et al., 2019	0.4	0.0794	479	-0.1	0.0629	391	4.4%	0.50 [0.49, 0.51]
Yoong et al., 2020	0.1	0.0814	262	0.05	0.0521	255	4.4%	0.05 [0.04, 0.06]
**Subtotal (95% CI)**			**999**			**874**	**13.1%**	**0.20 [-0.11, 0.50]**
Heterogeneity: Tau² = 0.07; Chi² = 6126.70, df = 2 (P < 0.00001); I² = 100%								
Test for overall effect: Z = 1.27 (P = 0.20)								
**22.1.5 Dairy food items and alternatives servings/day**								
Seward et al.; 2017	0.48	0.0541	258	0.45	0.063	288	4.4%	0.03 [0.02, 0.04]
Yoong et al., 2019	0.1	0.0784	479	-0.1	0.0814	391	4.4%	0.20 [0.19, 0.21]
Yoong et al., 2020	0.07	0.0773	262	-0.32	0.073	255	4.4%	0.39 [0.38, 0.40]
**Subtotal (95% CI)**			**999**			**934**	**13.1%**	**0.21 [0.01, 0.40]**
Heterogeneity: Tau² = 0.03; Chi² = 1915.46, df = 2 (P < 0.00001); I² = 100%								
Test for overall effect: Z = 2.05 (P = 0.04)								
**Total (95% CI)**			**9107**			**9469**	**100.0%**	**0.20 [0.12, 0.29]**
Heterogeneity: Tau² = 0.04; Chi² = 47521.65, df = 22 (P < 0.00001); I² = 100%								
Test for overall effect: Z = 4.97 (P < 0.00001)								
Test for subgroup differences: Chi² = 6.11, df = 4 (P = 0.19), I² = 34.6%								

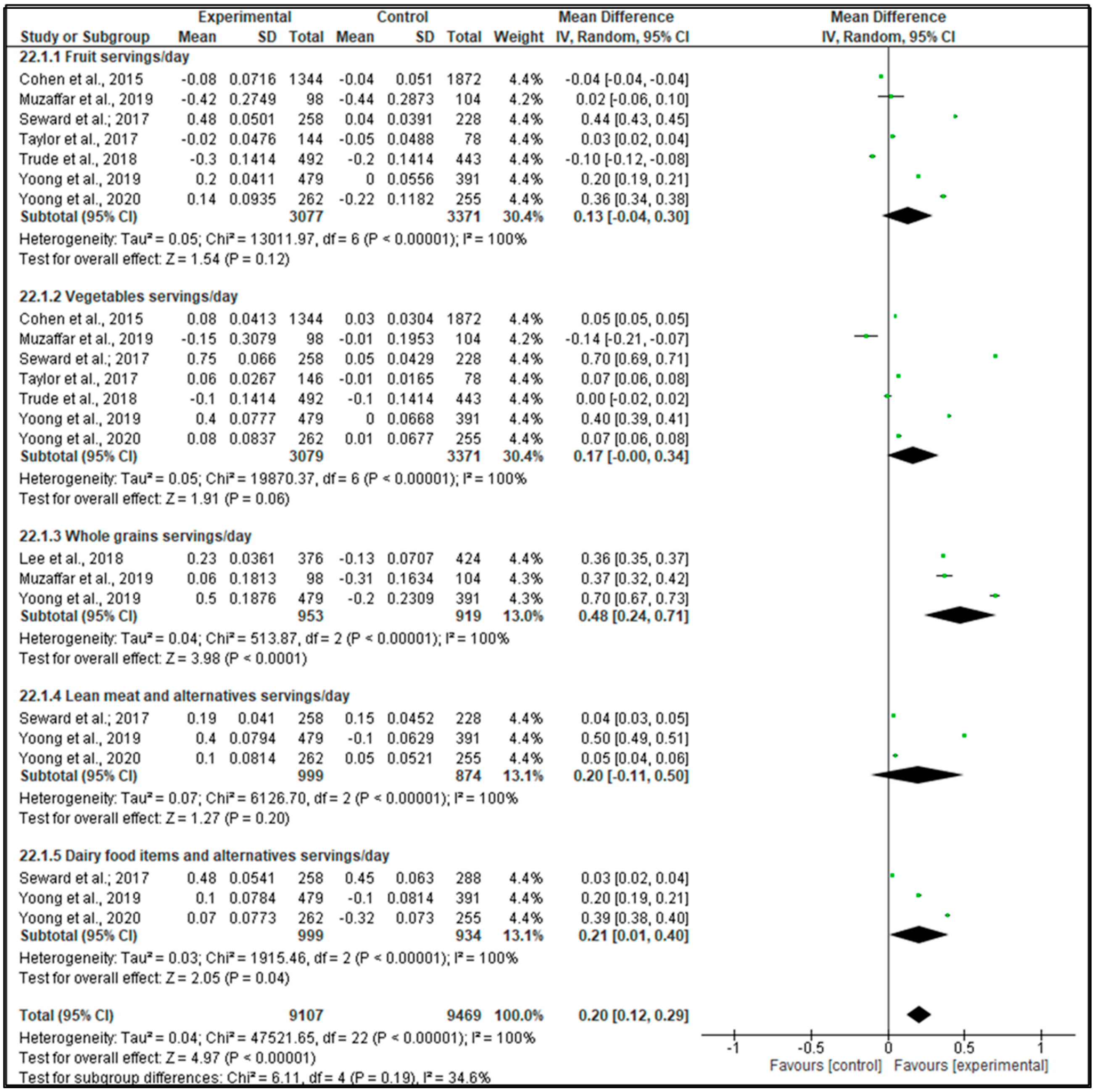

Forest plot of the effectiveness of increasing the dietary intake of healthy food items (servings/day), according to the included intervention studies (RCTs only).

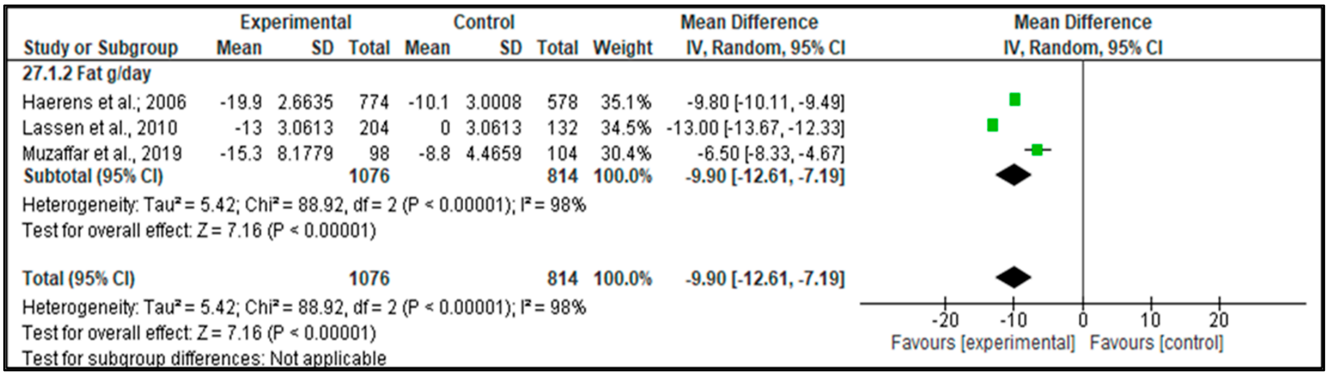

Forest plot of the effectiveness of decreasing the dietary intake of saturated fat, fat, and added sugar nutrients (g/day), according to the included intervention studies (RCTs only).
