# Supplementary material for: Interventions to Promote Healthy Meals in Full-Service Restaurants and Canteens: A Systematic Review and Meta-Analysis
Source: Nutrients. 2021 Apr 18;13(4):1350. doi: 10.3390/nu13041350 (PMC8073122; doi:10.3390/nu13041350)
Supplement: Supplementary file 1 [file nutrients-13-01350-s001.zip › Table S2_Included intervention study results.pdf]

Table S2: Included intervention study results.

Food availability				
**Study**	**Setting**	**Outcome**	**Tool**	**Mean difference from baseline to postintervention**
Beets et al.; 2016.	Community	Implementation of a healthy eating policy comprising changes in the types of snacks served at schools.	Direct observations.	Total F&V (days/week): IG +3.3 (p=0.006*); CG 0.0 (p=0.521); p<0.001*[‡]<br>Dips (days/week): IG +1.5 (p=0.007*); CG -0.2 (p=0.157); p<0.001*[‡]<br>Dairy unsweetened snacks (days/week): IG 0 (p=0.782); CG -0.1 (p=0.413); p=0.284[‡]<br>Dairy sweetened snacks (days/week): IG 0; CG -0.2; p=0.157[‡]<br>Salty unflavored snacks (days/week): IG -0.4 (p=0.918); CG -0.3 (p=0.912); p=0.011*[‡]<br>Salty flavored snacks (days/week): IG -1.4 (p=0.147); CG 0.0 (p=0.714); p=0.667[‡]<br>Desserts (days/week): IG -2.6 (p=0.013*); CG +0.1 (p=0.529); p=0.002*[‡]<br>Unsweetened cereals (days/week): IG +0.7 (p=0.482); CG +0.5 (p=0.084); p=0.804[‡]<br>Sugar-sweetened cereals (days/week): IG -0.9; CG -0.1; p=0.654[‡]<br>Total sugar-sweetened beverages (days/week): IG -2.3 (p=0.017*); CG +0.3 (p=0.221); p<0.005*[‡]<br>100% fruit juice (days/week): IG -0.1 (p=0.317); CG +0.4 (p=0.395); p=0.018*[‡]<br>Water (days/week): IG +1.8 (p=0.257); CG +0.2 (p=1.000); p=0.969[‡]<br>Unflavored milk (days/week): IG +0.6 (p=0.620); CG -0.4 (p=0.158); p=0.425[‡]
Cohen et al.; 2014.	School	Change in availability and quantity of WG and RG foods offered to students during breakfast and lunch.	Collection of production records, cafeteria recipes, product labels, and telephone interviews.	WG (options/breakfast): IG +0.4; CG +0.4; p=0.85[‡]<br>RG (options/breakfast): IG -0.2; CG -0.3; p=0.86[‡]<br>WG (options/lunch): IG +0.2; CG 0.0; p=0.06[‡]<br>RG (options/lunch): IG +0.1; CG 0.0; p=0.76[‡]<br>%days offering WG at breakfast (%days): IG +10; CG +10; p=N/A[‡]<br>%days offering RG at breakfast (%days): IG 0; CG -10; p=0.53[‡]<br>%days offering WG at lunch (%days): IG +10; CG +5; p=0.047*[‡]<br>%days offering RG at lunch (%days): IG 0; CG +5; p=0.26[‡]
Grady et al.; 2020.	School	Increase in the servings of food groups that comply with dietary guidelines on the menu.	Menu reviews.	Vegetables (servings/day): IG +0.32; CG +0.16; p=0.08[‡]<br>Fruit (servings/day): IG +0.21; CG -0.03; p<0.001*[‡]<br>Cereals and breads (servings/day): IG +0.15; CG +0.06; p=0.28[‡]<br>Meat and alternatives (servings/day): IG +0.15; CG +0.01; p=0.01*[‡]<br>Dairy and alternatives (servings/day): IG +0.04; CG -0.07; p=0.08[‡]<br>Discretionary (times/day): IG -0.39; CG -0.07; p=0.008*[‡]

Martínez-Donate et al.; 2015.	Community	Improve the food environment in restaurants and food stores.	Nutrition Environment Measurement Survey (NEMS).	NEMS-Restaurants (points/90): IG +10.7 (p=0.010*); CG +1.7 (p=0.596); p=N/A[ŧ]<br>NEMS-Food stores (points/66): IG -3.5; CG -2.5; p=N/A[ŧ]
Morshed et al.; 2016.	School	Changes in the food served at the child-care center canteen.	Direct observations.	Fruit (servings/day): IG -0.09; CG -0.15; p>0.05[ŧ]<br>Vegetables (servings/day): IG +0.22; CG +0.11; p>0.05[ŧ]<br>Whole grains (servings/day): IG +0.02; CG -0.09; p>0.05[ŧ]<br>Discretionary fat (grams/day): IG -0.32; CG -0.37; p>0.05[ŧ]<br>Added sugar (teaspoons/day): IG -0.35; CG -0.18; p>0.05[ŧ]<br>Milk fat (grams/day): IG -1.57; CG -0.13; p<0.05*[ŧ]
Nathan et al.; 2016.	School	Implementation of a healthy canteen policy comprising changes in the food items offered on the school menu.	Audits of the canteen menu.	Menus with no red or banned foods and beverages (*n*, %): IG +8 (+30.3); CG 0 (+0.33); p=0.002*[ŧ]<br>Menus with >50% of green food items (*n*, %): IG +9 (+34.3); CG -2 (-6.8); p=0.03*[ŧ]
Rosmawati et al.; 2017.	School	Change in the proportion of competitive foods served at the school canteen.	Direct observations.	Carbohydrate (%/day): IG -1.5; CG 0; p=0.170[ŧ]<br>Protein (%/day): IG +2.0; CG +0.5; p=0.243[ŧ]<br>High fat (%/day): IG +3.0; CG +1.5; p=0.915[ŧ]<br>Added sugar (%/day): IG 0; CG 0; p=0.746[ŧ]<br>Vegetable (%/day): IG +0.5; CG 0; p=0.158[ŧ]<br>Fruits (%/day): IG +0.5; CG 0; p=0.263[ŧ]<br>Forbidden food (%/day): IG +0.5; CG -0.5; p=0.158[ŧ]<br>Not recommended food (%/day): IG +2.5; CG 0; p=0.598[ŧ]<br>Fast foods (%/day): IG +3.0; CG 0; p=0.574[ŧ]<br>Milk and milk products (%/day): IG 0; CG -0.5; p=0.015*[ŧ]
Seward et al.; 2017.	School	Change in the number of food servings compliant with nutrition guideline recommendations.	Menu reviews.	Vegetables (servings/day): IG +0.98; CG +0.05; p<0.001*[ŧ]<br>Fruit (servings/day): IG +0.27; CG -0.13; p=0.002*[ŧ]<br>Breads and cereals (servings/day): IG +0.34; CG -0.04; p=0.045*[ŧ]<br>Meat and alternatives (servings/day): IG +0.22; CG +0.08; p=0.002*[ŧ]<br>Dairy (servings/day): IG +0.21; CG -0.09; p=0.019*[ŧ]<br>Discretionary (servings/day): IG -0.55; CG -0.07; p<0.001*[ŧ]

Souza et al.; 2014.	School	Change the availability of added sugar served at school.	Records of the monthly availability and use of the food items.	Sugar (Kg/child): IG -6,0; CG +3.4; p=0.21[ǂ]<br>Donuts (Kg/child): IG +3.7; CG +11.6; p=0.15[ǂ]<br>Milky coffee (Kg/child): IG -4.2; CG -7.9; p=0.55[ǂ]<br>Banana cereal (Kg/child): IG -1.8; CG -9.7; p=0.11[ǂ]<br>Chocolate cereal (Kg/child): IG -0.8; CG -1.8; p=0.77[ǂ]<br>Chocolate milk (Kg/child): IG +0.2; CG -1.1; p=0.43[ǂ]<br>Powdered milk (Kg/child): IG -3.1; CG -2.1; p=0.69[ǂ]<br>Cake mix (Kg/child): IG -0.03; CG -1.6; p=0.28[ǂ]
Story et al.; 2003.	School	Change in the nutrient content of school lunches.	Collection of menus, recipes, vendor products with labels, and nutrient information of prepared, processed, and packaged foods.	Total calories (kcal/lunch): IG +0.1; CG -30.2; p=0.87[ǂ]<br>Total fat (g/lunch): IG -3.5; CG -2; p=0.10[ǂ]<br>Energy from total fat (%/lunch): IG -4.8; CG 0; p=0.006*[ǂ]<br>Saturated fat (g/lunch): IG -2; CG -1.1; p=0.07[ǂ]<br>Energy from saturated fat (%/lunch): IG -2.7; CG -1; p=0.003*[ǂ]<br>Protein (g/lunch): IG 0; CG -1.2; p=0.31[ǂ]<br>Energy from protein (%/lunch): IG +0.1; CG 0; p=0.21[ǂ]<br>Carbohydrate (g/lunch): IG +8.1; CG -1.5; p=0.08[ǂ]<br>Energy from carbohydrate (%/lunch): IG +4.8; CG +1.4; p=0.01*[ǂ]<br>Total sugars (g/lunch): IG +2.6; CG +2.7; p=0.53[ǂ]<br>Sucrose (g/lunch): IG +0.9; CG +1.1; p=0.25[ǂ]<br>Dietary fiber (g/lunch): IG +1; CG -0.8; p=0.72[ǂ]<br>Sodium (mg/lunch): IG +78.9; CG -27.4; p=0.12[ǂ]
Wolfenden et al.; 2015.	Community	Increase the availability of fruit, vegetable, and non-sugar sweetened beverages in the club canteen.	Computer-Assisted Telephone Interviews (CATI), and self-reports.	F&V, *n*(%): IG +12 (+37%); CG +4 (+14%); p=0.006*[ǂ]<br>Non-sugar sweetened beverages, *n*(%): IG -10 (0); CG -15 (-3%); p=0.459[ǂ]<br>F&V promoted, *n*(%): IG +15 (+39.9%); CG -1 (-2.1%); p<0.001*[ǂ]
Wolfenden et al.; 2017.	School	Implementation of a healthy canteen policy comprising changes in the food items offered on the school menu.	Audits of the canteen menu.	Menus with no red or banned foods and beverages (*n*, %): IG +15 (+58.94%); CG -5 (-13.81%); p<0.01*[ǂ]<br>Menus with >50% of green items (*n*, %): IG +17 (+67.19%); CG +1 (+6.67%); p<0.01*[ǂ]

Yoong et al.; 2016.	School	Implementation of a healthy canteen policy comprising changes in the food items offered on the school menu.	Audits of the canteen menu.	Menus with no red or banned foods and beverages (*n*, %): IG +9 (+31.7%); CG 0 (+4.2%); p>0.05Ɨ<br>Menus with >50% of green items (*n*, %): IG +6 (+25.4%); CG 0 (+9.8%); p>0.05Ɨ<br>Red items on the menu, %: IG -6.1; CG +0.8; p<0.01*Ɨ<br>Green items on the menu, %: IG +12.6; CG +0.3; p>0.05Ɨ<br>Amber items on the menu, %: IG -4; CG -0.9; p>0.05Ɨ
Bell et al.; 2014.	School	Change the content of food and drinks provided to children by the service, as well as those packed for children by parents.	Computer-Assisted Telephone Interview (CATI), self-reports and telephone interviews.	High-fat, -salt and/or -sugar processed food (items/day): IG -0.9 (p<0.01*); CG -0.2 (p=0.09); p=0.001*Ɨ<br>Sweetened beverages (items/day): IG -0.4 (p<0.002*); CG -0.1 (p=0.29); p<0.001*Ɨ<br>Child-sized servings of fruits/day: IG -0.5 (p<0.002*); CG -0.1 (p=0.79); p=0.05*Ɨ<br>Child-sized servings of vegetables/day: IG +1.0 (p<0.001*); CG +0.2 (p=0.04*); p<0.001*Ɨ
**Dietary intake**				
**Study**	**Setting**	**Outcome**	**Tool**	**Mean difference from baseline to postintervention**
Anderson et al.; 2005.	School	Increase students' consumption of fruits and vegetables in the school canteen.	3-day food diary and dietary recall interviews.	Fruit (g/day): IG +50; CG +7; p=0.042*Ɨ<br>Vegetables (g/day): IG -17; CG -15; p=0.823Ɨ<br>F&V (g/day): IG +33; CG -7; p=0.617Ɨ<br>Energy (kJ/day): IG +4; CG -348; p=0.327Ɨ<br>Energy as fat (%/day): IG -0.5; CG -0.6; p=0.929Ɨ<br>Energy as carbohydrate (%/day): IG +0.5; CG +1.4; p=0.368Ɨ<br>Energy as protein (%/day): IG 0; CG -0.8; p=0.097Ɨ<br>Starch (g/day): IG +3; CG +3; p=0.980Ɨ<br>Sucrose (g/day): IG -0.5; CG -4; p=0.578Ɨ
Bogart et al.; 2011.	School	Improve students' healthy dietary intake.	Surveys.	Soda (% students consuming/day): IG -0.9; CG +6.7; p>0.05Ɨ<br>Sports/fruit drinks (% students consuming/day): IG -12; CG -6.6; p>0.05Ɨ

Cohen et al.; 2015.	School	Improve students' healthy dietary intake.	Plate waste method.	Entrée (%): IG-A -6.9; IG-B +8.0; IG-C +13.3; CG +6.7; p>0.05‡<br>Cups of fruits (servings/day): IG-A +0.23 (p<0.05*‡); IG-B -0.03 (p>0.05‡); IG-C -0.08 (p>0.05‡); CG -0.04<br>Cups of vegetables (servings/day): IG-A +0.16 (p<0.05*‡); IG-B +0.01 (p>0.05‡); IG-C +0.08 (p<0.05*‡); CG +0.03<br>Fruit (%): IG-A +36.4 (p>0.05‡); IG-B -4.1 (p>0.05‡); IG-C -8.2 (p>0.05‡); CG -2.2<br>Vegetable (%): IG-A +31 (p<0.05*‡); IG-B +0.5 (p>0.05‡); IG-C +14.5 (p<0.05*‡); CG +6.5
Habib-Mourad et al.; 2014.	School	Improve students' healthy dietary intake.	Questionnaires.	Chips, %(*n*): IG -28.2 (-55); CG -1.4 (-5); p<0.05*‡<br>Chocolate, %(*n*): IG -21.3 (-42); CG -15.5 (-30); p>0.05‡<br>Soft drinks, %(*n*): IG -17.4 (-34); CG -13.5 (-26); p<0.05*‡<br>Sweetened beverages, %(*n*): IG -20.6 (-42); CG +4.2 (+5); p>0.05‡<br>Fruit, %(*n*): IG -4.4 (-12); CG -6.7 (-15); p>0.05‡<br>Sandwich, %(*n*): IG 0 (-2); CG +1.2(0); p>0.05‡
Haerens et al.; 2006.	School	Improve students' healthy dietary intake.	Self-administered and Food Frequency Questionnaires (FFQ).	Fat (g/day): IG -2.7 (boys), -19.9 (girls); CG +1.3 (boys), -10.1 (girls); p>0.05‡ (boys), p<0.05*‡ (girls)<br>Energy from fat (%/day): IG -6.9 (boys), -9.2 (girls); CG -5 (boys), -4.9 (girls); p>0.05‡ (boys), p<0.001*‡ (girls)<br>Fruit (pieces/week): IG -0.2 (boys), +0.4 (girls); CG 0 (boys), -0.1 (girls); p>0.05‡ (boys and girls)<br>Soft drink (glass/day): IG +0.1 (boys), -0.5 (girls); CG +0.2 (boys), -0.3 (girls); p>0.05‡ (boys and girls)<br>Water (glass/day): IG +0.5 (boys), +0.3 (girls); CG +0.2 (boys), +0.4 (girls); p>0.05‡ (boys and girls)
Kenney et al.; 2015.	School	Increase students' water consumption during school lunches.	Direct observations.	Water (oz./student/lunch): IG +0.53; CG -0.06; p<0.001*‡<br>Free water (% students/lunch): IG +7.3; CG -2.0; p<0.001*‡<br>Sugar sweetened-beverages (% students/lunch): IG -1; CG +2.3; p<0.001*‡<br>Milk (% students/lunch): IG -0.8; CG -0.4; p=0.85‡<br>100% juice (% students/lunch): IG -0.5; CG +2.4; p=0.03*‡<br>Other beverages (% students/lunch): IG -0.2; CG -0.1; p=0.47‡

Lassen et al.; 2010.	Workplace	Improve employees' healthy dietary intake.	Dietary recording method (face to face interviews and self-administered food diaries).	Energy (kJ/day): IG -869 (p=0.003*); CG -266 (p=0.44); p=0.16ǂ<br>Fat (g/day): IG -13 (p<0.001*); CG 0 (p=0.99); p=0.007*ǂ<br>Saturated fat (g/day): IG -5 (p<0.001*); CG 0 (p=0.74); p=0.028*ǂ<br>Carbohydrate (%E/day): IG +1.2 (p=0.14); CG -1.9 (p=0.025*); p=0.010*ǂ<br>Fat (%E/day): IG -2.2 (p=0.002*); CG +1.5 (p=0.049*); p<0.001*ǂ<br>Protein (%E/day): IG +0.7 (p=0.02*); CG -0.2 (p=0.60); p=0.07ǂ<br>Added sugar (g/day): IG -8 (p=0.019*); CG -7 (p=0.049*); p=0.78ǂ<br>Added sugar (g/10MJ): IG -8 (p=0.002*); CG -6 (p=0.04*); p=0.68ǂ<br>Fiber (g/day): IG +1 (p=0.27); CG 0 (p=0.96); p=0.44ǂ<br>Fiber (g/10 MJ): IG +3 (p<0.001*); CG 0 (p=0.49); p=0.035*ǂ<br>F&V (g/day): IG +44 (p=0.07); CG +16 (p=0.48); p=0.41ǂ<br>F&V (g/10MJ): IG +95 (p=0.002*); CG +36 (p=0.22); p=0.25ǂ<br>Potatoes (g/day): IG +14 (p=0.03*); CG -10 (p=0.44); p=0.15ǂ<br>Potatoes (g/10MJ): IG +30 (p=0.005*); CG -15 (p=0.33); p=0.06ǂ<br>Cake and sweets (g/day): IG -19 (p=0.002*); CG +2 (p=0.82); p=0.037*ǂ<br>Cake and sweets (g/10 MJ): IG -18 (p=0.002*); CG +3 (p=0.65); p=0.032*ǂ
Lee et al.; 2018.	Community	Improve students' healthy dietary intake of foods and beverages served at snack time (on-site food services).	Plate waste method through digital photography.	100% juice (ounces/snack/day): IG -0.52; CG +1.90; p<0.0001*ǂ<br>Fruits and vegetables (servings/snack/day): IG +0.33; CG +0.01; p<0.0001*ǂ<br>Trans fats (servings/snack/day): IG -0.30; CG +0.01; p<0.0001*ǂ<br>Whole grains (servings/snack/day): IG +0.23; CG -0.13; p<0.0001*ǂ<br>Food calories (servings/snack/day): IG -107.8; CG -17.3; p<0.0001*ǂ<br>Beverage calories (servings/snack/day): IG -60; CG -5; p<0.0001*ǂ
Muzaffar et al.; 2019.	Community	Improve students' healthy dietary intake.	24-h dietary recall.	Total kcal/day: IG -250; CG -225; p=0.596ǂ<br>Fruits (servings/day): IG -0.42; CG -0.44; p=0.993ǂ<br>Vegetables (servings/day): IG -0.15; CG -0.01; p=0.880ǂ<br>Whole grains (servings/day): IG +0.06; CG -0.31; p=0.014*ǂ<br>Total fat (g/day): IG -15.3; CG -8.8; p=0.419ǂ<br>Total sugar (g/day): IG +0.3; CG -15.2; p=0.945ǂ<br>Total fiber (g/day): IG -0.24; CG -0.73; p=0.332ǂ<br>Total salt (mg/day): IG -624; CG -357; p=0.877ǂ

Ochoa-Avilés et al.; 2017.	School	Improve students' healthy dietary intake.	24-h dietary recall.	Fruit and vegetables (g/day): IG -54; CG -38.4; p=0.005*[ŧ]<br>Added sugar (g/day): IG -11.1; CG -6.1; p=0.006[ŧ]<br>Total fat (% E/day): IG +0.4; CG +0.9; p=0.25[ŧ]<br>Unhealthy snacking during snack time (g/day): IG -34.5; CG +0.6; p=0.04*[ŧ]<br>Unhealthy snacking at school (% consumers): IG +8.1; CG +11.7; p=0.49[ŧ]<br>Breakfast intake (% consumers): IG -4.4; CG +5.2; p=0.39[ŧ]
Seward et al.; 2017.	School	Improve students' healthy dietary intake.	Plate waste method.	Vegetables (servings/day): IG +0.75; CG +0.05; p=0.005*[ŧ]<br>Fruit (servings/day): IG +0.48; CG +0.04; p=0.042*[ŧ]<br>Breads and cereals (servings/day): IG +0.66; CG +0.21; p=0.661[ŧ]<br>Meat (servings/day): IG +0.19; CG +0.15; p=0.816[ŧ]<br>Dairy (servings/day): IG +0.48; CG +0.45; p=0.822[ŧ]<br>Discretionary (servings/day): IG -0.45; CG -0.04; p=0.136[ŧ]
Siega-Riz et al.; 2011.	School	Improve students' healthy dietary intake.	Block Kids Questionnaire (semi-quantified Food Frequency Questionnaire (FFQ)).	Energy (kcal/day): IG +12; CG +15; p=0.406[ŧ]<br>Carbohydrates (g/day): IG +5; CG +7; p=0.414[ŧ]<br>Protein (g/day): IG 0; CG -1; p=0.454[ŧ]<br>Fat (g/day): IG -1; CG -1; p=0.523[ŧ]<br>Fiber (g/day): IG -1; CG -1; p=0.571[ŧ]<br>Grains (g/day): IG +1; CG +2; p=0.704[ŧ]<br>Fruits (g/day): IG -17; CG -38; p=0.002*[ŧ]<br>Vegetables (g/day): IG -14; CG -21; p=0.895[ŧ]<br>Legumes (g/day): IG +1; CG +1; p=0.532[ŧ]<br>Sweets (g/day): IG 0; CG -1; p=0.349[ŧ]<br>Water (g/day): IG +117; CG +86; p=0.008*[ŧ]<br>Sweetened beverage (g/day): IG +54; CG +100; p=0.309[ŧ]<br>Fruit juice (g/day): IG -5; CG -6; p=0.782[ŧ]<br>2% fat and whole milk (g/day): IG +8; CG +4; p=0.616[ŧ]<br>1% fat milk (g/day): IG 0; CG +2; p=0.956[ŧ]

Souza et al.; 2014.	School	Improve the school cook's healthy dietary intake.	Food Frequency Questionnaire (FFQ).	Energy (kcal/day): IG -0.952; CG -0.982; p=0.88ᵻ<br>Carbohydrates (%/day): IG -1.1; CG -1.6; p=0.81ᵻ<br>Protein (%/day): IG +0.9; CG +0.8; p=0.90ᵻ<br>Lipid (%/day): IG +0.2; CG +0.8; p=0.70ᵻ<br>Total energy derived from sugar, sweets and sugary drinks (%E/day): IG +1.11; CG -0.65; p=0.36ᵻ<br>Added Sugar (portions/day): IG +0.3; CG -0.4; p=0.10ᵻ<br>Sweets (portions/day): IG -0.6; CG -0.2; p=0.18ᵻ<br>Sugary drinks (portions/day): IG -0.5; CG -1.8; p=0.07ᵻ
Taylor et al.; 2017.	School	Improve students' fruit and vegetable lunchtime intake.	Plate waste method through digital photography.	Fruit (cups/lunch): IG -0.02; CG -0.05; p=0.689ᵻ<br>Vegetable (cups/lunch): IG +0.06; CG -0.01; p= 0.032*ᵻ
Trude et al.; 2018.	Community	Improve children's intake of low-sugar foods and beverages.	Food Frequency Questionnaire (FFQ).	Caloric intake (kcal/day): IG -351.6 (9-12y), -300.8 (13-15y), -348.8 (total); CG -351.2 (9-12y), -489.8 (13-15y), -421.4 (total); p>0.05ᵻ<br>Sugary beverages (kcal/day): IG +55.1 (9-12y), +18.4 (13-15y); CG +29.3 (9-12y), +7.7 (13-15y); p>0.05ᵻ<br>Fruit punch (ounces/day): IG +1.3 (9-12y), +0.3 (13-15y); CG +0.3 (9-12y), +0.1 (13-15y); p>0.05ᵻ<br>Snacks (% kcal from sweets/day): IG -0.6 (9-12y), -0.4 (13-15y); CG -0.6 (9-12y), -2.4 (13-15y); p>0.05ᵻ for 9-12y and p<0.05*ᵻ for 13-15y<br>Total sugar (g/day): IG +1.6 (9-12y), -2.4 (13-15y); CG -0.1 (9-12y), -5.3 (13-15y); p>0.05ᵻ<br>Sodium (mg/day): IG +21.6 (9-12y), -18.2 (13-15y); CG +16.2 (9-12y), +12.5 (13-15y); p>0.05ᵻ<br>Fruit (cups/day): IG -0.5 (9-12y), -0.1 (13-15y), -0.3 (total); CG -0.3 (9-12y), +0.4 (13-15y), -0.2 (total); p>0.05ᵻ<br>Vegetable (cups/day): IG -0.2 (9-12y), 0 (13-15y), -0.1 (total); CG -0.2 (9-12y), -0.1 (13-15y), -0.1 (total); p>0.05ᵻ<br>Fat (servings/day): IG -0.1 (9-12y), -0.1 (13-15y); CG 0 (9-12y), 0 (13-15y); p>0.05ᵻ

Warren et al.; 2003.	School	Prevention of children obesity comprising changes in students' dietary intake.	24-h dietary recall, parental questionnaires, and Food Frequency Questionnaire (FFQ).	Vegetables (portion/frequency/week): IG-A +0.9; IG-B +0.2; IG-C +0.5; CG +0.1; p>0.05[Ɨ]<br>Salads (portion/frequency/week): IG-A +0.5; IG-B +0.4; IG-C +0.3; CG +1.3; p>0.05[Ɨ]<br>Fresh fruit (portion/frequency/week): IG-A +0.7*; IG-B +0.2; IG-C +0.6; CG +1.5*; p>0.05[Ɨ]<br>Other fruit[a] (portion/frequency/week): IG-A +0.4; IG-B +0.4; IG-C +0.4; CG +0.5; p>0.05[Ɨ]<br>Confectionery (portion/frequency/week): IG-A -0.4; IG-B 0; IG-C -0.1; CG -0.2; p>0.05[Ɨ]<br>Crisps (portion/frequency/week): IG-A -0.5; IG-B -0.4; IG-C 0; CG -0.2; p>0.05[Ɨ]
Yoong et al.; 2019.	School	Improve students' healthy dietary intake.	Questionnaires.	Fruit (servings/day): IG +0.2; CG 0; p=0.08[Ɨ]<br>Vegetable (servings/day): IG +0.4; CG 0; p<0.01*[Ɨ]<br>Whole grain cereals (servings/day): IG +0.5; CG -0.2; p<0.025*[Ɨ]<br>Dairy/dairy alternatives (servings/day): IG +0.1; CG -0.1; p=0.12[Ɨ]<br>Meat/meat alternatives (servings/day): IG +0.4; CG -0.1; p<0.01*[Ɨ]
Yoong et al.; 2020.	School	Improve students' healthy dietary intake.	Plate waste method.	Vegetables (servings/day): IG +0.08; CG +0.01; p=0.64[Ɨ]<br>Fruit (servings/day): IG +0.14; CG -0.22; p=0.003*[Ɨ]<br>Cereals and breads (servings/day): IG -0.4; CG +0.17; p<0.001*[Ɨ]<br>Meat and alternatives (servings/day): IG +0.1; CG +0.05; p=0.59[Ɨ]<br>Dairy and alternatives (servings/day): IG +0.07; CG -0.32; p<0.001*[Ɨ]<br>Discretionary (servings/day): IG -0.3; CG +0.07; p=0.002*[Ɨ]
Burgess-Champoux et al.; 2008.	School	Increase students' intake of whole grain.	Plate waste method.	WG (servings/lunch): IG +1.05 (p<0.05); CG +0.09; p=0.0001*[Ɨ]<br>RG (servings/lunch): IG -1.11*; CG -0.46*; p=0.001*[Ɨ]<br>Energy (kcal/lunch): IG +21; CG -35*; p=0.19[Ɨ]<br>Fiber (g/lunch): IG +1.3*; CG -0.4; p=0.001*[Ɨ]
Geaney et al.; 2016.	Workplace	Improve employees' healthy dietary intake.	24-h dietary recall.	Salt intake (g/day): IG-A -0.6 (p=0.144[Ɨ]); IG-B -0.5 (p=0.459[Ɨ]); IG-C -1.4 (p=0.010*[Ɨ]); GC +0.7<br>Total energy (kcal/day): IG-A -156.6 (p=0.173[Ɨ]); IG-B -110.8 (p=0.253[Ɨ]); IG-C -241.7 (p=0.440[Ɨ]); GC +26.5<br>Total fat (g/day): IG-A -7.1 (p=0.066[Ɨ]); IG-B -11.4 (p=0.986[Ɨ]); IG-C -14.2 (p=0.120[Ɨ]); GC +1.9<br>Total fat (E%/day): IG-A -0.6 (p=0.115[Ɨ]); IG-B -2.0 (p=0.338[Ɨ]); IG-C -2.2 (p=0.095[Ɨ]); GC +0.2<br>Saturated fat (g/day): IG-A -3.2 (p=0.034*[Ɨ]); IG-B -8.8 (p=0.261[Ɨ]); IG-C -7.0 (p=0.013*[Ɨ]);

				IG-C +1.8<br>Saturated fat (E%/day): IG-A -0.7 (p=0.053[‡]); IG-B -2.7 (p=0.017*[‡]); IG-C -1.6 (p=0.006*[‡]); GC +0.7<br>Total sugars (g/day): IG-A -6.8 (p=0.318[‡]); IG-B -4.6 (p=0.035*[‡]); IG-C -11.1 (p=0.601[‡]); GC +9.1<br>Fiber (g/day): IG-A -0.2 (p=0.923[‡]); IG-B -0.4 (p=0.510[‡]); IG-C +0.2 (p=0.071[‡]); GC +0.2
Quinn et al.; 2018.	School	Improve students' healthy dietary intake.	Visual quarter-waste method.	**Proportion of students among those who selected consuming/lunch**:<br>Fruit including juice: IG -0.02; CG +0.03; p=0.02*[‡]<br>Fruit excluding juice: IG +0.11; CG +0.11; p=0.89[‡]<br>Vegetables including potatoes: IG +0.04; CG +0.09; p=0.13[‡]<br>Vegetables excluding potatoes: IG +0.14; CG +0.15; p=0.70[‡]<br>Low-fat milk: IG +0.03; CG +0.07; p=0.61[‡]<br>**Items consumed among those who selected:**<br>Fruit including juice (number items/lunch): IG +0.04; CG +0.08; p=0.45[‡]<br>Fruit excluding juice (number items/lunch): IG +0.11; CG +0.29; p=0.03*[‡]<br>Vegetables including potatoes (number items/lunch): IG -0.03; CG +0.10; p=0.02*[‡]<br>Vegetables excluding potatoes (number items/lunch): IG 0; CG +0.15; p=0.19[‡]<br>Low-fat milk (number items/lunch): IG 0; CG +0.06; p=0.60[‡]
Williams et al.; 2002.	School	Decrease students' intake of saturated fat.	Plate waste method, telephone interviews, and food records.	Kcal/day: IG +74; CG +32; p>0.05[‡]<br>Fat (g/day): IG +0.8; CG +2.4; p>0.05[‡]<br>Saturated fat (g/day): IG -1.1; CG +1; p<0.01*[‡]<br>Fat (%kcal/day): IG -2.8; CG +4.3; p<0.001*[‡]<br>Saturated fat (%kcal/day): IG -3.0; CG +1.2; p<0.001*[‡]<br>Protein (g/day): IG +4.5; CG +2.4; p>0.05[‡]<br>Fiber (g/day): IG +0.1; CG -0.5; p>0.05[‡]
**Food purchase**				
**Study**	**Setting**	**Outcome**	**Tool**	**Mean difference from baseline to post-intervention**
Ayala et al.; 2017.	Community	Increase the purchase of new healthy child menu items.	Weekly sales data collection.	Total weekly sales of new child menus ($/week): IG -60; CG -28.7; p>0.05[‡]

Bogart et al.; 2014.	School	Increase healthy food served at the school cafeteria.	School cafeteria daily records and sales data.	Fruit (% servings/day): IG 0; CG -0.05 (p<0.05*); p<0.10‡<br>Vegetables (% servings/day): IG -0.03; CG -0.01; p>0.05‡<br>All lunches (% servings/day): IG -0.05 (p<0.001*); CG -0.08 (p<0.001*); p<0.01*‡<br>Free/reduced lunch (% servings/day): IG +0.02; CG -0.04 (p<0.001*); p<0.01*‡<br>Full-price lunch (% servings/day): IG 0; CG -0.02 (p<0.001*); p<0.001*‡<br>Snack sales (per student/day): IG -0.08 (p<0.001*); CG -0.05 (p<0.001*); p<0.01*‡
Cohen et al.; 2015.	School	Increase students' healthy purchases.	Plate waste method.	Entrée (% of students selecting): IG-A +0; IG-B +0; IG-C +0; CG +0; p=N/A<br>Fruit (% of students selecting): IG-A +24.7 (p<0.0001*‡); IG-B -4.5 (p<0.0001*‡); IG-C -1 (p<0.0001*‡); CG -6.4<br>Vegetable (% of students selecting): IG-A +2.2 (p<0.0001*‡); IG-B +7.1 (p<0.0001*‡); IG-C +26.7 (p<0.0001*‡); CG -6
Delaney et al.; 2017.	School	Change the content of students' online canteen purchases.	Online canteen system data collection and telephone interviews.	Energy (kJ/lunch): IG -598.03; CG -14.35; p<0.001*‡<br>Saturated fat (g/lunch): IG -2.77; CG -0.22; p<0.001*‡<br>Sugar (g/lunch): IG +0.50; CG -0.08; p=0.17‡<br>Sodium (mg/lunch): IG -261.08; CG -6.83; p<0.005*‡<br>Green items (% item/lunch): IG +24.64; CG +1.49; p<0.001*‡<br>Red items (% item/lunch): IG -7.07; CG -1.47; p<0.01*‡
Giles et al.; 2012.	Community	Change in the type of beverages served/day during snack time.	Direct observations.	Water (ounces/day): IG +3.7; CG +0.1; p=0.01*‡<br>100% juice (ounces/day): IG -0.9; CG +0.1; p=0.19‡<br>Milk (ounces/day): IG -2.3; CG +0.2; p=0.06‡<br>Beverages (kcal/day): IG -55.4; CG +5.6; p=0.03*‡<br>Water (times served/day): IG +0.6; CG +0.04; p=0.01*‡<br>100% juice (times served/day): IG -0.2; CG +0.04; p=0.12‡<br>Milk (times served/day): IG -0.3; CG +0.03; p=0.06‡
Habib-Mourad et al.; 2014.	School	Increase students' healthy purchases.	Questionnaires.	Chips, %(*n*): IG -16.6 (-32); CG -0.3 (-2); p=0.008*‡<br>Chocolate, %(*n*): IG -20.7 (-40); CG -6.1 (-13); p=0.003*‡<br>Soft drinks, %(*n*): IG -14.6 (-28); CG -4.4 (-9); p=0.001*‡<br>Sweetened beverages, %(*n*): IG -15.2 (-30); CG +3.6 (+4); p=N/A<br>Manoushe, %(*n*): IG -7.8 (-16); CG -11 (-22); p=0.52‡<br>Croissant, %(*n*): IG -16 (-31); CG +0.6 (0); p=0.157‡
Martínez-Donate et al.; 2015.	Community	Improve healthy food purchases in restaurants and food stores.	Interviewer-administered surveys to customers.	Restaurants (% orders): IG +6.1%; CG -11.9 %; p=0.094‡<br>Food stores (% purchase): IG -1.5%; CG +9.9%; p=0.299‡

Taylor et al.; 2017.	School	Improve students' lunchtime fruit and vegetable selection.	Plate waste method through digital photography.	Fruit (cups/lunch): IG -0.02; CG -0.03; p=0.824[ǂ]<br>Vegetable (cups/lunch): IG +0.07; CG +0.01; p=0.217[ǂ]
Thorndike et al.; 2016.	Workplace	Increase employees' purchases of green-labeled foods.	Sales data from the cash register.	Purchase of green-labelled food items (%): IG-A +1.8% (p=0.07[ǂ]); IG-B +2.2% (p=0.03*[ǂ]); CG +0.1%
Trude et al.; 2018.	Community	Increase students' purchase of low-sugar foods and beverages.	Child Impact Questionnaire (CIQ).	Healthy items (number items/week): IG +9.5 (9-12y), +6.1 (13-15y); CG +6.8 (9-12y), +7.4 (13-15y); p<0.05*[ǂ] (9-12y), p>0.05[ǂ] (13-15y)<br>Unhealthy items (number items/week): IG +6.7 (9-12y), +3.9 (13-15y); CG +5.2 (9-12y), +4.6 (13-15y); p<0.05*[ǂ] (9-12y), p>0.05[ǂ] (13-15y)
Webb et al.; 2011.	Workplace	Increase employees' purchase of low-calories entrées, side dishes, and snacks.	Sales data from electronic cash registers.	Healthy target entrées (% items/lunch): IG-A +0.028; CG +0.050; p=N/A<br>Healthy target side dishes (% items/lunch): IG +4.8; CG -4.8; p=0.0007*[ǂ]<br>Healthy target snacks (% items/lunch): IG +1.3; CG -8.1; p=0.006*[ǂ]
Wolfenden et al.; 2015.	Community	Increase the purchase of fruit, vegetable, and non-sugar sweetened beverages in the club canteen.	Computer-Assisted Telephone Interviews (CATI), and self-reports.	F&V, *n* (%) items: IG +56 (+11.4%); CG -4 (+1.1%); p=0.033*[ǂ]<br>Non-sugar sweetened beverages, *n* (%) items: IG +33 (+13.4%); CG -35 (+2.6%); p=0.015*[ǂ]
Wolfenden et al.; 2017.	School	Implementation of a healthy canteen policy comprising changes in the food items offered on the school menu.	Direct observations.	Energy (kJ/student purchase): -132.32 (difference at follow-up between IG and CG); p=0.08[ǂ]<br>Total fat (g/student purchase): -1.51 (difference at follow-up between IG and CG); p=0.03*[ǂ]<br>Sodium (mg/student purchase): -46.81 (difference at follow-up between IG and CG); p=0.07[ǂ]
Wyse et al.; 2019.	School	Increase students' online purchase of fruit and vegetable snacks.	Daily online lunches sales data.	% of all lunch orders containing fruit or vegetable snack food (%/lunch): IG +1.39; CG +0.75; p=0.490[ǂ]<br>% of all lunch order items that are fruit or vegetable snack food (%/lunch): IG +0.84; CG +0.37; p=0.991[ǂ]
Bogart et al.; 2011.	School	Improve students' purchase of fruits and healthy	Daily cafeteria sales data.	Fruits (% students served/day): IG +17.6; CG -4.9; p<0.001*[ǂ]<br>Healthy entrées (% students served/day): IG +1.9; CG -0.5; p<0.001*[ǂ]

		entrées.		
Quinn et al.; 2018.	School	Improve students' healthy purchases.	Direct observations.	Fruit including juice (proportion students selecting/lunch): IG +0.04; CG -0.05; p=0.004*[ƚ]<br>Fruit excluding juice (proportion students selecting/lunch): IG +0.12; CG -0.05; p<0.001*[ƚ]<br>Vegetables including potatoes (proportion students selecting/lunch): IG +0.28; CG +0.24; p=0.30[ƚ]<br>Vegetables excluding potatoes (proportion students selecting/lunch): IG +0.02; CG -0.05; p=0.11[ƚ]<br>Low-fat milk (proportion students selecting/lunch): IG +0.04; CG +0.02; p=0.55[ƚ]<br>Fruit including juice (number items/lunch): IG +0.16; CG -0.05; p=0.001*[ƚ]<br>Fruit excluding juice (number items/lunch): IG +0.15; CG -0.03; p<0.001*[ƚ]<br>Vegetables including potatoes (number items/lunch): IG +0.32; CG +0.29; p=0.89[ƚ]<br>Vegetables excluding potatoes (number items/lunch): IG +0.01; CG -0.05; p=0.24[ƚ]<br>Low-fat milk (number items/lunch): IG +0.04; CG +0.01; p=0.51[ƚ]

The included studies in the present systematic review are sorted in the following table by RCTs and Non-RCTs, and by alphabetical order.
F&V: Fruit and Vegetable; IG: Intervention Group; CG: Control Group; WG: Whole Grains; RG: Refined Grains; N/A: Not Available.
*Significant differences p≤0.05; [ƚ]Between groups comparison at postintervention.
