# Supplementary material for: Interventions to Promote Healthy Meals in Full-Service Restaurants and Canteens: A Systematic Review and Meta-Analysis
Source: Nutrients. 2021 Apr 18;13(4):1350. doi: 10.3390/nu13041350 (PMC8073122; doi:10.3390/nu13041350)
Supplement: Supplementary file 1 [file nutrients-13-01350-s001.zip › Table S3_Quality of the included intervention studies.pdf]

Table S3: Quality of the included intervention studies.

Included studies	Selection bias	Study design	Confounders	Blinding	Data collection methods	Withdrawals and dropouts	Quality
Ayala et al.; 2017.	Weak	Strong	Weak	Weak	Weak	Weak	WEAK
Anderson et al.; 2005.	Weak	Strong	Weak	Weak	Weak	Weak	WEAK
Beets et al.; 2016.	Weak	Strong	Moderate	Weak	Weak	Weak	WEAK
Bogart et al.; 2014.	Moderate	Strong	Weak	Weak	Weak	Moderate	WEAK
Cohen et al.; 2014.	Moderate	Strong	Weak	Weak	Weak	Weak	WEAK
Cohen et al.; 2015.	Weak	Strong	Weak	Weak	Weak	Weak	WEAK
Delaney et al.; 2017.	Weak	Strong	Weak	Weak	Weak	Strong	WEAK
Giles et al.; 2012.	Moderate	Strong	Moderate	Weak	Weak	Strong	WEAK
Grady et al.; 2020.	Moderate	Strong	Weak	Weak	Weak	Strong	WEAK
Habib-Mourad et al.; 2014.	Strong	Strong	Weak	Weak	Weak	Strong	WEAK

Haerens et al.; 2006.	Weak	Strong	Moderate	Weak	Moderate	Moderate	WEAK
Kenney et al.; 2015.	Moderate	Strong	Weak	Weak	Weak	Strong	WEAK
Lassen et al.; 2010.	Moderate	Strong	Moderate	Weak	Weak	Weak	WEAK
Lee et al.; 2018.	Moderate	Strong	Moderate	Weak	Weak	Moderate	WEAK
Martínez-Donate et al.; 2015.	Weak	Strong	Weak	Weak	Weak	Weak	WEAK
Morshed et al.; 2016.	Weak	Strong	Moderate	Weak	Weak	Weak	WEAK
Muzaffar et al.; 2019.	Weak	Strong	Weak	Weak	Weak	Weak	WEAK
Nathan et al.; 2016.	Weak	Strong	Weak	Weak	Moderate	Strong	WEAK
Ochoa-Avilés et al.; 2017.	Weak	Strong	Moderate	Weak	Weak	Moderate	WEAK
Rosmawati et al.; 2017.	Weak	Strong	Weak	Weak	Weak	Weak	WEAK
Seward et al.; 2017.	Moderate	Strong	Weak	Weak	Moderate	Moderate	WEAK
Siega-Riz et al.; 2011.	Strong	Strong	Moderate	Weak	Weak	Weak	WEAK

Souza et al.; 2014.	Strong	Strong	Moderate	Weak	Weak	Strong	WEAK
Story et al.; 2003.	Weak	Strong	Weak	Weak	Weak	Weak	WEAK
Taylor et al.; 2017.	Weak	Strong	Weak	Weak	Weak	Moderate	WEAK
Thorndike et al.; 2016.	Weak	Strong	Weak	Weak	Week	Strong	WEAK
Trude et al.; 2018.	Weak	Strong	Moderate	Weak	Weak	Moderate	WEAK
Warren et al.; 2003	Moderate	Strong	Weak	Weak	Moderate	Strong	WEAK
Webb et al.; 2011.	Weak	Strong	Weak	Weak	Weak	Weak	WEAK
Wolfenden et al.; 2015.	Weak	Strong	Weak	Weak	Weak	Strong	WEAK
Wolfenden et al.; 2017.	Strong	Strong	Moderate	Weak	Weak	Strong	WEAK
Wyse et al.; 2019.	Weak	Strong	Weak	Weak	Weak	Strong	WEAK
Yoong et al.; 2016.	Moderate	Strong	Moderate	Weak	Weak	Moderate	WEAK
Yoong et al.; 2019.	Moderate	Strong	Weak	Weak	Strong	Moderate	WEAK

Yoong et al.; 2020.	Moderate	Strong	Weak	Weak	Weak	Moderate	WEAK
Bell et al.; 2014.	Strong	Strong	Moderate	Weak	Weak	Weak	WEAK
Bogart et al.; 2011.	Weak	Strong	Weak	Weak	Weak	Moderate	WEAK
Burgess-Champoux et al.; 2008.	Weak	Strong	Weak	Weak	Moderate	Strong	WEAK
Geaney et al.; 2016.	Strong	Strong	Weak	Weak	Moderate	Moderate	WEAK
Quinn et al.; 2018.	Moderate	Strong	Moderate	Weak	Strong	Weak	WEAK
Williams et al.; 2002.	Weak	Strong	Weak	Weak	Weak	Weak	WEAK

The included studies in the present systematic review are sorted in the following table by RCTs and Non-RCTs, and by alphabetical order.
